# Measurement of the top quark mass in the all-jets final state at $\sqrt{s}=13\,\text {TeV}$ and combination with the lepton+jets channel

**DOI:** 10.1140/epjc/s10052-019-6788-2

**Published:** 2019-04-06

**Authors:** A. M. Sirunyan, A. Tumasyan, W. Adam, F. Ambrogi, E. Asilar, T. Bergauer, J. Brandstetter, M. Dragicevic, J. Erö, A. Escalante Del Valle, M. Flechl, R. Frühwirth, V. M. Ghete, J. Hrubec, M. Jeitler, N. Krammer, I. Krätschmer, D. Liko, T. Madlener, I. Mikulec, N. Rad, H. Rohringer, J. Schieck, R. Schöfbeck, M. Spanring, D. Spitzbart, W. Waltenberger, J. Wittmann, C.-E. Wulz, M. Zarucki, V. Chekhovsky, V. Mossolov, J. Suarez Gonzalez, E. A. De Wolf, D. Di Croce, X. Janssen, J. Lauwers, A. Lelek, M. Pieters, H. Van Haevermaet, P. Van Mechelen, N. Van Remortel, S. Abu Zeid, F. Blekman, J. D’Hondt, J. De Clercq, K. Deroover, G. Flouris, D. Lontkovskyi, S. Lowette, I. Marchesini, S. Moortgat, L. Moreels, Q. Python, K. Skovpen, S. Tavernier, W. Van Doninck, P. Van Mulders, I. Van Parijs, D. Beghin, B. Bilin, H. Brun, B. Clerbaux, G. De Lentdecker, H. Delannoy, B. Dorney, G. Fasanella, L. Favart, A. Grebenyuk, A. K. Kalsi, T. Lenzi, J. Luetic, N. Postiau, E. Starling, L. Thomas, C. Vander Velde, P. Vanlaer, D. Vannerom, Q. Wang, T. Cornelis, D. Dobur, A. Fagot, M. Gul, I. Khvastunov, D. Poyraz, C. Roskas, D. Trocino, M. Tytgat, W. Verbeke, B. Vermassen, M. Vit, N. Zaganidis, H. Bakhshiansohi, O. Bondu, G. Bruno, C. Caputo, P. David, C. Delaere, M. Delcourt, A. Giammanco, G. Krintiras, V. Lemaitre, A. Magitteri, K. Piotrzkowski, A. Saggio, M. Vidal Marono, P. Vischia, J. Zobec, F. L. Alves, G. A. Alves, G. Correia Silva, C. Hensel, A. Moraes, M. E. Pol, P. Rebello Teles, E. Belchior Batista Das Chagas, W. Carvalho, J. Chinellato, E. Coelho, E. M. Da Costa, G. G. Da Silveira, D. De Jesus Damiao, C. De Oliveira Martins, S. Fonseca De Souza, H. Malbouisson, D. Matos Figueiredo, M. Melo De Almeida, C. Mora Herrera, L. Mundim, H. Nogima, W. L. Prado Da Silva, L. J. Sanchez Rosas, A. Santoro, A. Sznajder, M. Thiel, E. J. Tonelli Manganote, F. Torres Da Silva De Araujo, A. Vilela Pereira, S. Ahuja, C. A. Bernardes, L. Calligaris, T. R. Fernandez Perez Tomei, E. M. Gregores, P. G. Mercadante, S. F. Novaes, SandraS. Padula, A. Aleksandrov, R. Hadjiiska, P. Iaydjiev, A. Marinov, M. Misheva, M. Rodozov, M. Shopova, G. Sultanov, A. Dimitrov, L. Litov, B. Pavlov, P. Petkov, W. Fang, X. Gao, L. Yuan, M. Ahmad, J. G. Bian, G. M. Chen, H. S. Chen, M. Chen, Y. Chen, C. H. Jiang, D. Leggat, H. Liao, Z. Liu, S. M. Shaheen, A. Spiezia, J. Tao, E. Yazgan, H. Zhang, S. Zhang, J. Zhao, Y. Ban, G. Chen, A. Levin, J. Li, L. Li, Q. Li, Y. Mao, S. J. Qian, D. Wang, Y. Wang, C. Avila, A. Cabrera, C. A. Carrillo Montoya, L. F. Chaparro Sierra, C. Florez, C. F. González Hernández, M. A. Segura Delgado, B. Courbon, N. Godinovic, D. Lelas, I. Puljak, T. Sculac, Z. Antunovic, M. Kovac, V. Brigljevic, D. Ferencek, K. Kadija, B. Mesic, M. Roguljic, A. Starodumov, T. Susa, M. W. Ather, A. Attikis, M. Kolosova, G. Mavromanolakis, J. Mousa, C. Nicolaou, F. Ptochos, P. A. Razis, H. Rykaczewski, M. Finger, M. Finger, E. Ayala, E. Carrera Jarrin, H. Abdalla, S. Khalil, A. Mohamed, S. Bhowmik, A. Carvalho Antunes De Oliveira, R. K. Dewanjee, K. Ehataht, M. Kadastik, M. Raidal, C. Veelken, P. Eerola, H. Kirschenmann, J. Pekkanen, M. Voutilainen, J. Havukainen, J. K. Heikkilä, T. Järvinen, V. Karimäki, R. Kinnunen, T. Lampén, K. Lassila-Perini, S. Laurila, S. Lehti, T. Lindén, P. Luukka, T. Mäenpää, H. Siikonen, E. Tuominen, J. Tuominiemi, T. Tuuva, M. Besancon, F. Couderc, M. Dejardin, D. Denegri, J. L. Faure, F. Ferri, S. Ganjour, A. Givernaud, P. Gras, G. Hamel de Monchenault, P. Jarry, C. Leloup, E. Locci, J. Malcles, G. Negro, J. Rander, A. Rosowsky, M. Ö. Sahin, M. Titov, A. Abdulsalam, C. Amendola, I. Antropov, F. Beaudette, P. Busson, C. Charlot, R. Granier de Cassagnac, I. Kucher, A. Lobanov, J. Martin Blanco, C. Martin Perez, M. Nguyen, C. Ochando, G. Ortona, P. Paganini, J. Rembser, R. Salerno, J. B. Sauvan, Y. Sirois, A. G. Stahl Leiton, A. Zabi, A. Zghiche, J.-L. Agram, J. Andrea, D. Bloch, G. Bourgatte, J.-M. Brom, E. C. Chabert, V Cherepanov, C. Collard, E. Conte, J.-C. Fontaine, D. Gelé, U. Goerlach, M. Jansová, A.-C. Le Bihan, N. Tonon, P. Van Hove, S. Gadrat, S. Beauceron, C. Bernet, G. Boudoul, N. Chanon, R. Chierici, D. Contardo, P. Depasse, H. El Mamouni, J. Fay, L. Finco, S. Gascon, M. Gouzevitch, G. Grenier, B. Ille, F. Lagarde, I. B. Laktineh, H. Lattaud, M. Lethuillier, L. Mirabito, S. Perries, A. Popov, V. Sordini, G. Touquet, M. Vander Donckt, S. Viret, A. Khvedelidze, Z. Tsamalaidze, C. Autermann, L. Feld, M. K. Kiesel, K. Klein, M. Lipinski, M. Preuten, M. P. Rauch, C. Schomakers, J. Schulz, M. Teroerde, B. Wittmer, A. Albert, M. Erdmann, S. Erdweg, T. Esch, R. Fischer, S. Ghosh, A. Güth, T. Hebbeker, C. Heidemann, K. Hoepfner, H. Keller, L. Mastrolorenzo, M. Merschmeyer, A. Meyer, P. Millet, S. Mukherjee, T. Pook, M. Radziej, H. Reithler, M. Rieger, A. Schmidt, D. Teyssier, S. Thüer, G. Flügge, O. Hlushchenko, T. Kress, T. Müller, A. Nehrkorn, A. Nowack, C. Pistone, O. Pooth, D. Roy, H. Sert, A. Stahl, M. Aldaya Martin, T. Arndt, C. Asawatangtrakuldee, I. Babounikau, K. Beernaert, O. Behnke, U. Behrens, A. Bermúdez Martínez, D. Bertsche, A. A. Bin Anuar, K. Borras, V. Botta, A. Campbell, P. Connor, C. Contreras-Campana, V. Danilov, A. De Wit, M. M. Defranchis, C. Diez Pardos, D. Domínguez Damiani, G. Eckerlin, T. Eichhorn, A. Elwood, E. Eren, E. Gallo, A. Geiser, J. M. Grados Luyando, A. Grohsjean, M. Guthoff, M. Haranko, A. Harb, H. Jung, M. Kasemann, J. Keaveney, C. Kleinwort, J. Knolle, D. Krücker, W. Lange, T. Lenz, J. Leonard, K. Lipka, W. Lohmann, R. Mankel, I.-A. Melzer-Pellmann, A. B. Meyer, M. Meyer, M. Missiroli, G. Mittag, J. Mnich, V. Myronenko, S. K. Pflitsch, D. Pitzl, A. Raspereza, A. Saibel, M. Savitskyi, P. Saxena, P. Schütze, C. Schwanenberger, R. Shevchenko, A. Singh, H. Tholen, O. Turkot, A. Vagnerini, M. Van De Klundert, G. P. Van Onsem, R. Walsh, Y. Wen, K. Wichmann, C. Wissing, O. Zenaiev, R. Aggleton, S. Bein, L. Benato, A. Benecke, T. Dreyer, A. Ebrahimi, E. Garutti, D. Gonzalez, P. Gunnellini, J. Haller, A. Hinzmann, A. Karavdina, G. Kasieczka, R. Klanner, R. Kogler, N. Kovalchuk, S. Kurz, V. Kutzner, J. Lange, D. Marconi, J. Multhaup, M. Niedziela, C. E. N. Niemeyer, D. Nowatschin, A. Perieanu, A. Reimers, O. Rieger, C. Scharf, P. Schleper, S. Schumann, J. Schwandt, J. Sonneveld, H. Stadie, G. Steinbrück, F. M. Stober, M. Stöver, B. Vormwald, I. Zoi, M. Akbiyik, C. Barth, M. Baselga, S. Baur, E. Butz, R. Caspart, T. Chwalek, F. Colombo, W. De Boer, A. Dierlamm, K. El Morabit, N. Faltermann, B. Freund, M. Giffels, M. A. Harrendorf, F. Hartmann, S. M. Heindl, U. Husemann, I. Katkov, S. Kudella, S. Mitra, M. U. Mozer, Th. Müller, M. Musich, M. Plagge, G. Quast, K. Rabbertz, M. Schröder, I. Shvetsov, H. J. Simonis, R. Ulrich, S. Wayand, M. Weber, T. Weiler, C. Wöhrmann, R. Wolf, G. Anagnostou, G. Daskalakis, T. Geralis, A. Kyriakis, D. Loukas, G. Paspalaki, A. Agapitos, G. Karathanasis, P. Kontaxakis, A. Panagiotou, I. Papavergou, N. Saoulidou, K. Vellidis, K. Kousouris, I. Papakrivopoulos, G. Tsipolitis, I. Evangelou, C. Foudas, P. Gianneios, P. Katsoulis, P. Kokkas, S. Mallios, N. Manthos, I. Papadopoulos, E. Paradas, J. Strologas, F. A. Triantis, D. Tsitsonis, M. Bartók, M. Csanad, N. Filipovic, P. Major, M. I. Nagy, G. Pasztor, O. Surányi, G. I. Veres, G. Bencze, C. Hajdu, D. Horvath, Á. Hunyadi, F. Sikler, T. Á. Vámi, V. Veszpremi, G. Vesztergombi, N. Beni, S. Czellar, J. Karancsi, A. Makovec, J. Molnar, Z. Szillasi, P. Raics, Z. L. Trocsanyi, B. Ujvari, S. Choudhury, J. R. Komaragiri, P. C. Tiwari, S. Bahinipati, C. Kar, P. Mal, K. Mandal, A. Nayak, S. Roy Chowdhury, D. K. Sahoo, S. K. Swain, S. Bansal, S. B. Beri, V. Bhatnagar, S. Chauhan, R. Chawla, N. Dhingra, R. Gupta, A. Kaur, M. Kaur, S. Kaur, P. Kumari, M. Lohan, M. Meena, A. Mehta, K. Sandeep, S. Sharma, J. B. Singh, A. K. Virdi, G. Walia, A. Bhardwaj, B. C. Choudhary, R. B. Garg, M. Gola, S. Keshri, Ashok Kumar, S. Malhotra, M. Naimuddin, P. Priyanka, K. Ranjan, Aashaq Shah, R. Sharma, R. Bhardwaj, M. Bharti, R. Bhattacharya, S. Bhattacharya, U. Bhawandeep, D. Bhowmik, S. Dey, S. Dutt, S. Dutta, S. Ghosh, M. Maity, K. Mondal, S. Nandan, A. Purohit, P. K. Rout, A. Roy, G. Saha, S. Sarkar, T. Sarkar, M. Sharan, B. Singh, S. Thakur, P. K. Behera, A. Muhammad, R. Chudasama, D. Dutta, V. Jha, V. Kumar, D. K. Mishra, P. K. Netrakanti, L. M. Pant, P. Shukla, P. Suggisetti, T. Aziz, M. A. Bhat, S. Dugad, G. B. Mohanty, N. Sur, RavindraKumar Verma, S. Banerjee, S. Bhattacharya, S. Chatterjee, P. Das, M. Guchait, Sa. Jain, S. Karmakar, S. Kumar, G. Majumder, K. Mazumdar, N. Sahoo, S. Chauhan, S. Dube, V. Hegde, A. Kapoor, K. Kothekar, S. Pandey, A. Rane, A. Rastogi, S. Sharma, S. Chenarani, E. Eskandari Tadavani, S. M. Etesami, M. Khakzad, M. Mohammadi Najafabadi, M. Naseri, F. Rezaei Hosseinabadi, B. Safarzadeh, M. Zeinali, M. Felcini, M. Grunewald, M. Abbrescia, C. Calabria, A. Colaleo, D. Creanza, L. Cristella, N. De Filippis, M. De Palma, A. Di Florio, F. Errico, L. Fiore, A. Gelmi, G. Iaselli, M. Ince, S. Lezki, G. Maggi, M. Maggi, G. Miniello, S. My, S. Nuzzo, A. Pompili, G. Pugliese, R. Radogna, A. Ranieri, G. Selvaggi, A. Sharma, L. Silvestris, R. Venditti, P. Verwilligen, G. Abbiendi, C. Battilana, D. Bonacorsi, L. Borgonovi, S. Braibant-Giacomelli, R. Campanini, P. Capiluppi, A. Castro, F. R. Cavallo, S. S. Chhibra, G. Codispoti, M. Cuffiani, G. M. Dallavalle, F. Fabbri, A. Fanfani, E. Fontanesi, P. Giacomelli, C. Grandi, L. Guiducci, F. Iemmi, S. Lo Meo, S. Marcellini, G. Masetti, A. Montanari, F. L. Navarria, A. Perrotta, F. Primavera, A. M. Rossi, T. Rovelli, G. P. Siroli, N. Tosi, S. Albergo, A. Di Mattia, R. Potenza, A. Tricomi, C. Tuve, G. Barbagli, K. Chatterjee, V. Ciulli, C. Civinini, R. D’Alessandro, E. Focardi, G. Latino, P. Lenzi, M. Meschini, S. Paoletti, L. Russo, G. Sguazzoni, D. Strom, L. Viliani, L. Benussi, S. Bianco, F. Fabbri, D. Piccolo, F. Ferro, R. Mulargia, E. Robutti, S. Tosi, A. Benaglia, A. Beschi, F. Brivio, V. Ciriolo, S. Di Guida, M. E. Dinardo, S. Fiorendi, S. Gennai, A. Ghezzi, P. Govoni, M. Malberti, S. Malvezzi, D. Menasce, F. Monti, L. Moroni, M. Paganoni, D. Pedrini, S. Ragazzi, T. Tabarelli de Fatis, D. Zuolo, S. Buontempo, N. Cavallo, A. De Iorio, A. Di Crescenzo, F. Fabozzi, F. Fienga, G. Galati, A. O. M. Iorio, L. Lista, S. Meola, P. Paolucci, C. Sciacca, E. Voevodina, P. Azzi, N. Bacchetta, D. Bisello, A. Boletti, A. Bragagnolo, R. Carlin, P. Checchia, M. Dall’Osso, P. De Castro Manzano, T. Dorigo, U. Dosselli, F. Gasparini, U. Gasparini, A. Gozzelino, S. Y. Hoh, S. Lacaprara, P. Lujan, M. Margoni, A. T. Meneguzzo, J. Pazzini, M. Presilla, P. Ronchese, R. Rossin, F. Simonetto, A. Tiko, E. Torassa, M. Tosi, M. Zanetti, P. Zotto, G. Zumerle, A. Braghieri, A. Magnani, P. Montagna, S. P. Ratti, V. Re, M. Ressegotti, C. Riccardi, P. Salvini, I. Vai, P. Vitulo, M. Biasini, G. M. Bilei, C. Cecchi, D. Ciangottini, L. Fanò, P. Lariccia, R. Leonardi, E. Manoni, G. Mantovani, V. Mariani, M. Menichelli, A. Rossi, A. Santocchia, D. Spiga, K. Androsov, P. Azzurri, G. Bagliesi, L. Bianchini, T. Boccali, L. Borrello, R. Castaldi, M. A. Ciocci, R. Dell’Orso, G. Fedi, F. Fiori, L. Giannini, A. Giassi, M. T. Grippo, F. Ligabue, E. Manca, G. Mandorli, A. Messineo, F. Palla, A. Rizzi, G. Rolandi, P. Spagnolo, R. Tenchini, G. Tonelli, A. Venturi, P. G. Verdini, L. Barone, F. Cavallari, M. Cipriani, D. Del Re, E. Di Marco, M. Diemoz, S. Gelli, E. Longo, B. Marzocchi, P. Meridiani, G. Organtini, F. Pandolfi, R. Paramatti, F. Preiato, S. Rahatlou, C. Rovelli, F. Santanastasio, N. Amapane, R. Arcidiacono, S. Argiro, M. Arneodo, N. Bartosik, R. Bellan, C. Biino, A. Cappati, N. Cartiglia, F. Cenna, S. Cometti, M. Costa, R. Covarelli, N. Demaria, B. Kiani, C. Mariotti, S. Maselli, E. Migliore, V. Monaco, E. Monteil, M. Monteno, M. M. Obertino, L. Pacher, N. Pastrone, M. Pelliccioni, G. L. Pinna Angioni, A. Romero, M. Ruspa, R. Sacchi, R. Salvatico, K. Shchelina, V. Sola, A. Solano, D. Soldi, A. Staiano, S. Belforte, V. Candelise, M. Casarsa, F. Cossutti, A. Da Rold, G. Della Ricca, F. Vazzoler, A. Zanetti, D. H. Kim, G. N. Kim, M. S. Kim, J. Lee, S. Lee, S. W. Lee, C. S. Moon, Y. D. Oh, S. I. Pak, S. Sekmen, D. C. Son, Y. C. Yang, H. Kim, D. H. Moon, G. Oh, B. Francois, J. Goh, T. J. Kim, S. Cho, S. Choi, Y. Go, D. Gyun, S. Ha, B. Hong, Y. Jo, K. Lee, K. S. Lee, S. Lee, J. Lim, S. K. Park, Y. Roh, H. S. Kim, J. Almond, J. Kim, J. S. Kim, H. Lee, K. Lee, K. Nam, S. B. Oh, B. C. Radburn-Smith, S. h. Seo, U. K. Yang, H. D. Yoo, G. B. Yu, D. Jeon, H. Kim, J. H. Kim, J. S. H. Lee, I. C. Park, Y. Choi, C. Hwang, J. Lee, I. Yu, V. Veckalns, V. Dudenas, A. Juodagalvis, J. Vaitkus, Z. A. Ibrahim, M. A. B. Md Ali, F. Mohamad Idris, W. A. T. Wan Abdullah, M. N. Yusli, Z. Zolkapli, J. F. Benitez, A. Castaneda Hernandez, J. A. Murillo Quijada, H. Castilla-Valdez, E. De La Cruz-Burelo, M. C. Duran-Osuna, I. Heredia-De La Cruz, R. Lopez-Fernandez, J. Mejia Guisao, R. I. Rabadan-Trejo, M. Ramirez-Garcia, G. Ramirez-Sanchez, R. Reyes-Almanza, A. Sanchez-Hernandez, S. Carrillo Moreno, C. Oropeza Barrera, F. Vazquez Valencia, J. Eysermans, I. Pedraza, H. A. Salazar Ibarguen, C. Uribe Estrada, A. Morelos Pineda, D. Krofcheck, S. Bheesette, P. H. Butler, A. Ahmad, M. Ahmad, M. I. Asghar, Q. Hassan, H. R. Hoorani, W. A. Khan, M. A. Shah, M. Shoaib, M. Waqas, H. Bialkowska, M. Bluj, B. Boimska, T. Frueboes, M. Górski, M. Kazana, M. Szleper, P. Traczyk, P. Zalewski, K. Bunkowski, A. Byszuk, K. Doroba, A. Kalinowski, M. Konecki, J. Krolikowski, M. Misiura, M. Olszewski, A. Pyskir, M. Walczak, M. Araujo, P. Bargassa, C. Beirão Da Cruz E Silva, A. Di Francesco, P. Faccioli, B. Galinhas, M. Gallinaro, J. Hollar, N. Leonardo, J. Seixas, G. Strong, O. Toldaiev, J. Varela, S. Afanasiev, P. Bunin, M. Gavrilenko, I. Golutvin, I. Gorbunov, A. Kamenev, V. Karjavine, A. Lanev, A. Malakhov, V. Matveev, P. Moisenz, V. Palichik, V. Perelygin, S. Shmatov, S. Shulha, N. Skatchkov, V. Smirnov, N. Voytishin, A. Zarubin, V. Golovtsov, Y. Ivanov, V. Kim, E. Kuznetsova, P. Levchenko, V. Murzin, V. Oreshkin, I. Smirnov, D. Sosnov, V. Sulimov, L. Uvarov, S. Vavilov, A. Vorobyev, Yu. Andreev, A. Dermenev, S. Gninenko, N. Golubev, A. Karneyeu, M. Kirsanov, N. Krasnikov, A. Pashenkov, A. Shabanov, D. Tlisov, A. Toropin, V. Epshteyn, V. Gavrilov, N. Lychkovskaya, V. Popov, I. Pozdnyakov, G. Safronov, A. Spiridonov, A. Stepennov, V. Stolin, M. Toms, E. Vlasov, A. Zhokin, T. Aushev, R. Chistov, M. Danilov, D. Philippov, E. Tarkovskii, V. Andreev, M. Azarkin, I. Dremin, M. Kirakosyan, A. Terkulov, A. Baskakov, A. Belyaev, E. Boos, V. Bunichev, M. Dubinin, L. Dudko, V. Klyukhin, O. Kodolova, N. Korneeva, I. Lokhtin, S. Obraztsov, M. Perfilov, V. Savrin, A. Barnyakov, V. Blinov, T. Dimova, L. Kardapoltsev, Y. Skovpen, I. Azhgirey, I. Bayshev, S. Bitioukov, V. Kachanov, A. Kalinin, D. Konstantinov, P. Mandrik, V. Petrov, R. Ryutin, S. Slabospitskii, A. Sobol, S. Troshin, N. Tyurin, A. Uzunian, A. Volkov, A. Babaev, S. Baidali, V. Okhotnikov, P. Adzic, P. Cirkovic, D. Devetak, M. Dordevic, P. Milenovic, J. Milosevic, J. Alcaraz Maestre, A. Álvarez Fernández, I. Bachiller, M. Barrio Luna, J. A. Brochero Cifuentes, M. Cerrada, N. Colino, B. De La Cruz, A. Delgado Peris, C. Fernandez Bedoya, J. P. Fernández Ramos, J. Flix, M. C. Fouz, O. Gonzalez Lopez, S. Goy Lopez, J. M. Hernandez, M. I. Josa, D. Moran, A. Pérez-Calero Yzquierdo, J. Puerta Pelayo, I. Redondo, L. Romero, S. Sánchez Navas, M. S. Soares, A. Triossi, C. Albajar, J. F. de Trocóniz, J. Cuevas, C. Erice, J. Fernandez Menendez, S. Folgueras, I. Gonzalez Caballero, J. R. González Fernández, E. Palencia Cortezon, V. Rodríguez Bouza, S. Sanchez Cruz, J. M. Vizan Garcia, I. J. Cabrillo, A. Calderon, B. Chazin Quero, J. Duarte Campderros, M. Fernandez, P. J. Fernández Manteca, A. García Alonso, J. Garcia-Ferrero, G. Gomez, A. Lopez Virto, J. Marco, C. Martinez Rivero, P. Martinez Ruiz del Arbol, F. Matorras, J. Piedra Gomez, C. Prieels, T. Rodrigo, A. Ruiz-Jimeno, L. Scodellaro, N. Trevisani, I. Vila, R. Vilar Cortabitarte, N. Wickramage, D. Abbaneo, B. Akgun, E. Auffray, G. Auzinger, P. Baillon, A. H. Ball, D. Barney, J. Bendavid, M. Bianco, A. Bocci, C. Botta, E. Brondolin, T. Camporesi, M. Cepeda, G. Cerminara, E. Chapon, Y. Chen, G. Cucciati, D. d’Enterria, A. Dabrowski, N. Daci, V. Daponte, A. David, A. De Roeck, N. Deelen, M. Dobson, M. Dünser, N. Dupont, A. Elliott-Peisert, F. Fallavollita, D. Fasanella, G. Franzoni, J. Fulcher, W. Funk, D. Gigi, A. Gilbert, K. Gill, F. Glege, M. Gruchala, M. Guilbaud, D. Gulhan, J. Hegeman, C. Heidegger, V. Innocente, G. M. Innocenti, A. Jafari, P. Janot, O. Karacheban, J. Kieseler, A. Kornmayer, M. Krammer, C. Lange, P. Lecoq, C. Lourenço, L. Malgeri, M. Mannelli, A. Massironi, F. Meijers, J. A. Merlin, S. Mersi, E. Meschi, F. Moortgat, M. Mulders, J. Ngadiuba, S. Nourbakhsh, S. Orfanelli, L. Orsini, F. Pantaleo, L. Pape, E. Perez, M. Peruzzi, A. Petrilli, G. Petrucciani, A. Pfeiffer, M. Pierini, F. M. Pitters, D. Rabady, A. Racz, T. Reis, M. Rovere, H. Sakulin, C. Schäfer, C. Schwick, M. Selvaggi, A. Sharma, P. Silva, P. Sphicas, A. Stakia, J. Steggemann, D. Treille, A. Tsirou, A. Vartak, M. Verzetti, W. D. Zeuner, L. Caminada, K. Deiters, W. Erdmann, R. Horisberger, Q. Ingram, H. C. Kaestli, D. Kotlinski, U. Langenegger, T. Rohe, S. A. Wiederkehr, M. Backhaus, L. Bäni, P. Berger, N. Chernyavskaya, G. Dissertori, M. Dittmar, M. Donegà, C. Dorfer, T. A. Gómez Espinosa, C. Grab, D. Hits, T. Klijnsma, W. Lustermann, R. A. Manzoni, M. Marionneau, M. T. Meinhard, F. Micheli, P. Musella, F. Nessi-Tedaldi, F. Pauss, G. Perrin, L. Perrozzi, S. Pigazzini, M. Reichmann, C. Reissel, D. Ruini, D. A. Sanz Becerra, M. Schönenberger, L. Shchutska, V. R. Tavolaro, K. Theofilatos, M. L. Vesterbacka Olsson, R. Wallny, D. H. Zhu, T. K. Aarrestad, C. Amsler, D. Brzhechko, M. F. Canelli, A. De Cosa, R. Del Burgo, S. Donato, C. Galloni, T. Hreus, B. Kilminster, S. Leontsinis, I. Neutelings, G. Rauco, P. Robmann, D. Salerno, K. Schweiger, C. Seitz, Y. Takahashi, S. Wertz, A. Zucchetta, T. H. Doan, R. Khurana, C. M. Kuo, W. Lin, A. Pozdnyakov, S. S. Yu, P. Chang, Y. Chao, K. F. Chen, P. H. Chen, W.-S. Hou, Y. F. Liu, R.-S. Lu, E. Paganis, A. Psallidas, A. Steen, B. Asavapibhop, N. Srimanobhas, N. Suwonjandee, A. Bat, F. Boran, S. Cerci, S. Damarseckin, Z. S. Demiroglu, F. Dolek, C. Dozen, I. Dumanoglu, G. Gokbulut, Y. Guler, E. Gurpinar, I. Hos, C. Isik, E. E. Kangal, O. Kara, A. Kayis Topaksu, U. Kiminsu, M. Oglakci, G. Onengut, K. Ozdemir, S. Ozturk, D. Sunar Cerci, B. Tali, U. G. Tok, S. Turkcapar, I. S. Zorbakir, C. Zorbilmez, B. Isildak, G. Karapinar, M. Yalvac, M. Zeyrek, I. O. Atakisi, E. Gülmez, M. Kaya, O. Kaya, S. Ozkorucuklu, S. Tekten, E. A. Yetkin, M. N. Agaras, A. Cakir, K. Cankocak, Y. Komurcu, S. Sen, B. Grynyov, L. Levchuk, F. Ball, J. J. Brooke, D. Burns, E. Clement, D. Cussans, O. Davignon, H. Flacher, J. Goldstein, G. P. Heath, H. F. Heath, L. Kreczko, D. M. Newbold, S. Paramesvaran, B. Penning, T. Sakuma, D. Smith, V. J. Smith, J. Taylor, A. Titterton, K. W. Bell, A. Belyaev, C. Brew, R. M. Brown, D. Cieri, D. J. A. Cockerill, J. A. Coughlan, K. Harder, S. Harper, J. Linacre, K. Manolopoulos, E. Olaiya, D. Petyt, T. Schuh, C. H. Shepherd-Themistocleous, A. Thea, I. R. Tomalin, T. Williams, W. J. Womersley, R. Bainbridge, P. Bloch, J. Borg, S. Breeze, O. Buchmuller, A. Bundock, D. Colling, P. Dauncey, G. Davies, M. Della Negra, R. Di Maria, P. Everaerts, G. Hall, G. Iles, T. James, M. Komm, C. Laner, L. Lyons, A.-M. Magnan, S. Malik, A. Martelli, J. Nash, A. Nikitenko, V. Palladino, M. Pesaresi, D. M. Raymond, A. Richards, A. Rose, E. Scott, C. Seez, A. Shtipliyski, G. Singh, M. Stoye, T. Strebler, S. Summers, A. Tapper, K. Uchida, T. Virdee, N. Wardle, D. Winterbottom, J. Wright, S. C. Zenz, J. E. Cole, P. R. Hobson, A. Khan, P. Kyberd, C. K. Mackay, A. Morton, I. D. Reid, L. Teodorescu, S. Zahid, K. Call, J. Dittmann, K. Hatakeyama, H. Liu, C. Madrid, B. McMaster, N. Pastika, C. Smith, R. Bartek, A. Dominguez, A. Buccilli, S. I. Cooper, C. Henderson, P. Rumerio, C. West, D. Arcaro, T. Bose, D. Gastler, S. Girgis, D. Pinna, C. Richardson, J. Rohlf, L. Sulak, D. Zou, G. Benelli, B. Burkle, X. Coubez, D. Cutts, M. Hadley, J. Hakala, U. Heintz, J. M. Hogan, K. H. M. Kwok, E. Laird, G. Landsberg, J. Lee, Z. Mao, M. Narain, S. Sagir, R. Syarif, E. Usai, D. Yu, R. Band, C. Brainerd, R. Breedon, D. Burns, M. Calderon De La Barca Sanchez, M. Chertok, J. Conway, R. Conway, P. T. Cox, R. Erbacher, C. Flores, G. Funk, W. Ko, O. Kukral, R. Lander, M. Mulhearn, D. Pellett, J. Pilot, S. Shalhout, M. Shi, D. Stolp, D. Taylor, K. Tos, M. Tripathi, Z. Wang, F. Zhang, M. Bachtis, C. Bravo, R. Cousins, A. Dasgupta, S. Erhan, A. Florent, J. Hauser, M. Ignatenko, N. Mccoll, S. Regnard, D. Saltzberg, C. Schnaible, V. Valuev, E. Bouvier, K. Burt, R. Clare, J. W. Gary, S. M. A. Ghiasi Shirazi, G. Hanson, G. Karapostoli, E. Kennedy, F. Lacroix, O. R. Long, M. Olmedo Negrete, M. I. Paneva, W. Si, L. Wang, H. Wei, S. Wimpenny, B. R. Yates, J. G. Branson, P. Chang, S. Cittolin, M. Derdzinski, R. Gerosa, D. Gilbert, B. Hashemi, A. Holzner, D. Klein, G. Kole, V. Krutelyov, J. Letts, M. Masciovecchio, S. May, D. Olivito, S. Padhi, M. Pieri, V. Sharma, M. Tadel, J. Wood, F. Würthwein, A. Yagil, G. Zevi Della Porta, N. Amin, R. Bhandari, C. Campagnari, M. Citron, V. Dutta, M. Franco Sevilla, L. Gouskos, R. Heller, J. Incandela, H. Mei, A. Ovcharova, H. Qu, J. Richman, D. Stuart, I. Suarez, S. Wang, J. Yoo, D. Anderson, A. Bornheim, J. M. Lawhorn, N. Lu, H. B. Newman, T. Q. Nguyen, J. Pata, M. Spiropulu, J. R. Vlimant, R. Wilkinson, S. Xie, Z. Zhang, R. Y. Zhu, M. B. Andrews, T. Ferguson, T. Mudholkar, M. Paulini, M. Sun, I. Vorobiev, M. Weinberg, J. P. Cumalat, W. T. Ford, F. Jensen, A. Johnson, E. MacDonald, T. Mulholland, R. Patel, A. Perloff, K. Stenson, K. A. Ulmer, S. R. Wagner, J. Alexander, J. Chaves, Y. Cheng, J. Chu, A. Datta, K. Mcdermott, N. Mirman, J. R. Patterson, D. Quach, A. Rinkevicius, A. Ryd, L. Skinnari, L. Soffi, S. M. Tan, Z. Tao, J. Thom, J. Tucker, P. Wittich, M. Zientek, S. Abdullin, M. Albrow, M. Alyari, G. Apollinari, A. Apresyan, A. Apyan, S. Banerjee, L. A. T. Bauerdick, A. Beretvas, J. Berryhill, P. C. Bhat, K. Burkett, J. N. Butler, A. Canepa, G. B. Cerati, H. W. K. Cheung, F. Chlebana, M. Cremonesi, J. Duarte, V. D. Elvira, J. Freeman, Z. Gecse, E. Gottschalk, L. Gray, D. Green, S. Grünendahl, O. Gutsche, J. Hanlon, R. M. Harris, S. Hasegawa, J. Hirschauer, Z. Hu, B. Jayatilaka, S. Jindariani, M. Johnson, U. Joshi, B. Klima, M. J. Kortelainen, B. Kreis, S. Lammel, D. Lincoln, R. Lipton, M. Liu, T. Liu, J. Lykken, K. Maeshima, J. M. Marraffino, D. Mason, P. McBride, P. Merkel, S. Mrenna, S. Nahn, V. O’Dell, K. Pedro, C. Pena, O. Prokofyev, G. Rakness, F. Ravera, A. Reinsvold, L. Ristori, A. Savoy-Navarro, B. Schneider, E. Sexton-Kennedy, A. Soha, W. J. Spalding, L. Spiegel, S. Stoynev, J. Strait, N. Strobbe, L. Taylor, S. Tkaczyk, N. V. Tran, L. Uplegger, E. W. Vaandering, C. Vernieri, M. Verzocchi, R. Vidal, M. Wang, H. A. Weber, D. Acosta, P. Avery, P. Bortignon, D. Bourilkov, A. Brinkerhoff, L. Cadamuro, A. Carnes, D. Curry, R. D. Field, S. V. Gleyzer, B. M. Joshi, J. Konigsberg, A. Korytov, K. H. Lo, P. Ma, K. Matchev, N. Menendez, G. Mitselmakher, D. Rosenzweig, K. Shi, D. Sperka, J. Wang, S. Wang, X. Zuo, Y. R. Joshi, S. Linn, A. Ackert, T. Adams, A. Askew, S. Hagopian, V. Hagopian, K. F. Johnson, T. Kolberg, G. Martinez, T. Perry, H. Prosper, A. Saha, C. Schiber, R. Yohay, M. M. Baarmand, V. Bhopatkar, S. Colafranceschi, M. Hohlmann, D. Noonan, M. Rahmani, T. Roy, M. Saunders, F. Yumiceva, M. R. Adams, L. Apanasevich, D. Berry, R. R. Betts, R. Cavanaugh, X. Chen, S. Dittmer, O. Evdokimov, C. E. Gerber, D. A. Hangal, D. J. Hofman, K. Jung, J. Kamin, C. Mills, M. B. Tonjes, N. Varelas, H. Wang, X. Wang, Z. Wu, J. Zhang, M. Alhusseini, B. Bilki, W. Clarida, K. Dilsiz, S. Durgut, R. P. Gandrajula, M. Haytmyradov, V. Khristenko, J.-P. Merlo, A. Mestvirishvili, A. Moeller, J. Nachtman, H. Ogul, Y. Onel, F. Ozok, A. Penzo, C. Snyder, E. Tiras, J. Wetzel, B. Blumenfeld, A. Cocoros, N. Eminizer, D. Fehling, L. Feng, A. V. Gritsan, W. T. Hung, P. Maksimovic, J. Roskes, U. Sarica, M. Swartz, M. Xiao, A. Al-bataineh, P. Baringer, A. Bean, S. Boren, J. Bowen, A. Bylinkin, J. Castle, S. Khalil, A. Kropivnitskaya, D. Majumder, W. Mcbrayer, M. Murray, C. Rogan, S. Sanders, E. Schmitz, J. D. Tapia Takaki, Q. Wang, S. Duric, A. Ivanov, K. Kaadze, D. Kim, Y. Maravin, D. R. Mendis, T. Mitchell, A. Modak, A. Mohammadi, F. Rebassoo, D. Wright, A. Baden, O. Baron, A. Belloni, S. C. Eno, Y. Feng, C. Ferraioli, N. J. Hadley, S. Jabeen, G. Y. Jeng, R. G. Kellogg, J. Kunkle, A. C. Mignerey, S. Nabili, F. Ricci-Tam, M. Seidel, Y. H. Shin, A. Skuja, S. C. Tonwar, K. Wong, D. Abercrombie, B. Allen, V. Azzolini, A. Baty, R. Bi, S. Brandt, W. Busza, I. A. Cali, M. D’Alfonso, Z. Demiragli, G. Gomez Ceballos, M. Goncharov, P. Harris, D. Hsu, M. Hu, Y. Iiyama, M. Klute, D. Kovalskyi, Y.-J. Lee, P. D. Luckey, B. Maier, A. C. Marini, C. Mcginn, C. Mironov, S. Narayanan, X. Niu, C. Paus, D. Rankin, C. Roland, G. Roland, Z. Shi, G. S. F. Stephans, K. Sumorok, K. Tatar, D. Velicanu, J. Wang, T. W. Wang, B. Wyslouch, A. C. Benvenuti, R. M. Chatterjee, A. Evans, P. Hansen, J. Hiltbrand, Sh. Jain, S. Kalafut, M. Krohn, Y. Kubota, Z. Lesko, J. Mans, R. Rusack, M. A. Wadud, J. G. Acosta, S. Oliveros, E. Avdeeva, K. Bloom, D. R. Claes, C. Fangmeier, F. Golf, R. Gonzalez Suarez, R. Kamalieddin, I. Kravchenko, J. Monroy, J. E. Siado, G. R. Snow, B. Stieger, A. Godshalk, C. Harrington, I. Iashvili, A. Kharchilava, C. Mclean, D. Nguyen, A. Parker, S. Rappoccio, B. Roozbahani, G. Alverson, E. Barberis, C. Freer, Y. Haddad, A. Hortiangtham, G. Madigan, D. M. Morse, T. Orimoto, A. Tishelman-charny, T. Wamorkar, B. Wang, A. Wisecarver, D. Wood, S. Bhattacharya, J. Bueghly, O. Charaf, T. Gunter, K. A. Hahn, N. Odell, M. H. Schmitt, K. Sung, M. Trovato, M. Velasco, R. Bucci, N. Dev, R. Goldouzian, M. Hildreth, K. Hurtado Anampa, C. Jessop, D. J. Karmgard, K. Lannon, W. Li, N. Loukas, N. Marinelli, F. Meng, C. Mueller, Y. Musienko, M. Planer, R. Ruchti, P. Siddireddy, G. Smith, S. Taroni, M. Wayne, A. Wightman, M. Wolf, A. Woodard, J. Alimena, L. Antonelli, B. Bylsma, L. S. Durkin, S. Flowers, B. Francis, C. Hill, W. Ji, T. Y. Ling, W. Luo, B. L. Winer, S. Cooperstein, P. Elmer, J. Hardenbrook, N. Haubrich, S. Higginbotham, A. Kalogeropoulos, S. Kwan, D. Lange, M. T. Lucchini, J. Luo, D. Marlow, K. Mei, I. Ojalvo, J. Olsen, C. Palmer, P. Piroué, J. Salfeld-Nebgen, D. Stickland, C. Tully, S. Malik, S. Norberg, A. Barker, V. E. Barnes, S. Das, L. Gutay, M. Jones, A. W. Jung, A. Khatiwada, B. Mahakud, D. H. Miller, N. Neumeister, C. C. Peng, S. Piperov, H. Qiu, J. F. Schulte, J. Sun, F. Wang, R. Xiao, W. Xie, T. Cheng, J. Dolen, N. Parashar, Z. Chen, K. M. Ecklund, S. Freed, F. J. M. Geurts, M. Kilpatrick, Arun Kumar, W. Li, B. P. Padley, R. Redjimi, J. Roberts, J. Rorie, W. Shi, Z. Tu, A. Zhang, A. Bodek, P. de Barbaro, R. Demina, Y. t. Duh, J. L. Dulemba, C. Fallon, T. Ferbel, M. Galanti, A. Garcia-Bellido, J. Han, O. Hindrichs, A. Khukhunaishvili, E. Ranken, P. Tan, R. Taus, B. Chiarito, J. P. Chou, Y. Gershtein, E. Halkiadakis, A. Hart, M. Heindl, E. Hughes, S. Kaplan, R. Kunnawalkam Elayavalli, S. Kyriacou, I. Laflotte, A. Lath, R. Montalvo, K. Nash, M. Osherson, H. Saka, S. Salur, S. Schnetzer, D. Sheffield, S. Somalwar, R. Stone, S. Thomas, P. Thomassen, H. Acharya, A. G. Delannoy, J. Heideman, G. Riley, S. Spanier, O. Bouhali, A. Celik, M. Dalchenko, M. De Mattia, A. Delgado, S. Dildick, R. Eusebi, J. Gilmore, T. Huang, T. Kamon, S. Luo, D. Marley, R. Mueller, D. Overton, L. Perniè, D. Rathjens, A. Safonov, N. Akchurin, J. Damgov, F. De Guio, P. R. Dudero, S. Kunori, K. Lamichhane, S. W. Lee, T. Mengke, S. Muthumuni, T. Peltola, S. Undleeb, I. Volobouev, Z. Wang, A. Whitbeck, S. Greene, A. Gurrola, R. Janjam, W. Johns, C. Maguire, A. Melo, H. Ni, K. Padeken, F. Romeo, P. Sheldon, S. Tuo, J. Velkovska, M. Verweij, Q. Xu, M. W. Arenton, P. Barria, B. Cox, R. Hirosky, M. Joyce, A. Ledovskoy, H. Li, C. Neu, T. Sinthuprasith, Y. Wang, E. Wolfe, F. Xia, R. Harr, P. E. Karchin, N. Poudyal, J. Sturdy, P. Thapa, S. Zaleski, J. Buchanan, C. Caillol, D. Carlsmith, S. Dasu, I. De Bruyn, L. Dodd, B. Gomber, M. Grothe, M. Herndon, A. Hervé, U. Hussain, P. Klabbers, A. Lanaro, K. Long, R. Loveless, T. Ruggles, A. Savin, V. Sharma, N. Smith, W. H. Smith, N. Woods, CMS Collaboration

**Affiliations:** 1 Yerevan Physics Institute, Yerevan, Armenia; 2 Institut für Hochenergiephysik, Wien, Austria; 3 Institute for Nuclear Problems, Minsk, Belarus; 4 Universiteit Antwerpen, Antwerpen, Belgium; 5 Vrije Universiteit Brussel, Brussel, Belgium; 6 Université Libre de Bruxelles, Bruxelles, Belgium; 7 Ghent University, Ghent, Belgium; 8 Université Catholique de Louvain, Louvain-la-Neuve, Belgium; 9 Centro Brasileiro de Pesquisas Fisicas, Rio de Janeiro, Brazil; 10 Universidade do Estado do Rio de Janeiro, Rio de Janeiro, Brazil; 11 Universidade Estadual Paulista, Universidade Federal do ABC, São Paulo, Brazil; 12 Institute for Nuclear Research and Nuclear Energy, Bulgarian Academy of Sciences, Sofia, Bulgaria; 13 University of Sofia, Sofia, Bulgaria; 14 Beihang University, Beijing, China; 15 Institute of High Energy Physics, Beijing, China; 16 State Key Laboratory of Nuclear Physics and Technology, Peking University, Beijing, China; 17 Tsinghua University, Beijing, China; 18 Universidad de Los Andes, Bogota, Colombia; 19 Faculty of Electrical Engineering, Mechanical Engineering and Naval Architecture, University of Split, Split, Croatia; 20 Faculty of Science, University of Split, Split, Croatia; 21 Institute Rudjer Boskovic, Zagreb, Croatia; 22 University of Cyprus, Nicosia, Cyprus; 23 Charles University, Prague, Czech Republic; 24 Escuela Politecnica Nacional, Quito, Ecuador; 25 Universidad San Francisco de Quito, Quito, Ecuador; 26 Academy of Scientific Research and Technology of the Arab Republic of Egypt, Egyptian Network of High Energy Physics, Cairo, Egypt; 27 National Institute of Chemical Physics and Biophysics, Tallinn, Estonia; 28 Department of Physics, University of Helsinki, Helsinki, Finland; 29 Helsinki Institute of Physics, Helsinki, Finland; 30 Lappeenranta University of Technology, Lappeenranta, Finland; 31 IRFU, CEA, Université Paris-Saclay, Gif-sur-Yvette, France; 32 Laboratoire Leprince-Ringuet, Ecole polytechnique, CNRS/IN2P3, Université Paris-Saclay, Palaiseau, France; 33 Université de Strasbourg, CNRS, IPHC UMR 7178, Strasbourg, France; 34 Centre de Calcul de l’Institut National de Physique Nucleaire et de Physique des Particules, CNRS/IN2P3, Villeurbanne, France; 35 Université de Lyon, Université Claude Bernard Lyon 1, CNRS-IN2P3, Institut de Physique Nucléaire de Lyon, Villeurbanne, France; 36 Georgian Technical University, Tbilisi, Georgia; 37 Tbilisi State University, Tbilisi, Georgia; 38 RWTH Aachen University, I. Physikalisches Institut, Aachen, Germany; 39 RWTH Aachen University, III. Physikalisches Institut A, Aachen, Germany; 40 RWTH Aachen University, III. Physikalisches Institut B, Aachen, Germany; 41 Deutsches Elektronen-Synchrotron, Hamburg, Germany; 42 University of Hamburg, Hamburg, Germany; 43 Karlsruher Institut fuer Technologie, Karlsruhe, Germany; 44 Institute of Nuclear and Particle Physics (INPP), NCSR Demokritos, Aghia Paraskevi, Greece; 45 National and Kapodistrian University of Athens, Athens, Greece; 46 National Technical University of Athens, Athens, Greece; 47 University of Ioánnina, Ioánnina, Greece; 48 MTA-ELTE Lendület CMS Particle and Nuclear Physics Group, Eötvös Loránd University, Budapest, Hungary; 49 Wigner Research Centre for Physics, Budapest, Hungary; 50 Institute of Nuclear Research ATOMKI, Debrecen, Hungary; 51 Institute of Physics, University of Debrecen, Debrecen, Hungary; 52 Indian Institute of Science (IISc), Bangalore, India; 53 National Institute of Science Education and Research, HBNI, Bhubaneswar, India; 54 Panjab University, Chandigarh, India; 55 University of Delhi, Delhi, India; 56 Saha Institute of Nuclear Physics, HBNI, Kolkata, India; 57 Indian Institute of Technology Madras, Madras, India; 58 Bhabha Atomic Research Centre, Mumbai, India; 59 Tata Institute of Fundamental Research-A, Mumbai, India; 60 Tata Institute of Fundamental Research-B, Mumbai, India; 61 Indian Institute of Science Education and Research (IISER), Pune, India; 62 Institute for Research in Fundamental Sciences (IPM), Tehran, Iran; 63 University College Dublin, Dublin, Ireland; 64 INFN Sezione di Bari, Università di Bari, Politecnico di Bari, Bari, Italy; 65 INFN Sezione di Bologna, Università di Bologna, Bologna, Italy; 66 INFN Sezione di Catania, Università di Catania, Catania, Italy; 67 INFN Sezione di Firenze, Università di Firenze, Firenze, Italy; 68 INFN Laboratori Nazionali di Frascati, Frascati, Italy; 69 INFN Sezione di Genova, Università di Genova, Genova, Italy; 70 INFN Sezione di Milano-Bicocca, Università di Milano-Bicocca, Milano, Italy; 71 INFN Sezione di Napoli, Università di Napoli ‘Federico II’ , Napoli, Italy, Università della Basilicata, Potenza, Italy, Università G. Marconi, Roma, Italy; 72 INFN Sezione di Padova, Università di Padova, Padova, Italy, Università di Trento, Trento, Italy; 73 INFN Sezione di Pavia, Università di Pavia, Pavia, Italy; 74 INFN Sezione di Perugia, Università di Perugia, Perugia, Italy; 75 INFN Sezione di Pisa, Università di Pisa, Scuola Normale Superiore di Pisa, Pisa, Italy; 76 INFN Sezione di Roma, Sapienza Università di Roma, Rome, Italy; 77 INFN Sezione di Torino, Università di Torino, Torino, Italy, Università del Piemonte Orientale, Novara, Italy; 78 INFN Sezione di Trieste, Università di Trieste, Trieste, Italy; 79 Kyungpook National University, Daegu, Korea; 80 Institute for Universe and Elementary Particles, Chonnam National University, Kwangju, Korea; 81 Hanyang University, Seoul, Korea; 82 Korea University, Seoul, Korea; 83 Sejong University, Seoul, Korea; 84 Seoul National University, Seoul, Korea; 85 University of Seoul, Seoul, Korea; 86 Sungkyunkwan University, Suwon, Korea; 87 Riga Technical University, Riga, Latvia; 88 Vilnius University, Vilnius, Lithuania; 89 National Centre for Particle Physics, Universiti Malaya, Kuala Lumpur, Malaysia; 90 Universidad de Sonora (UNISON), Hermosillo, Mexico; 91 Centro de Investigacion y de Estudios Avanzados del IPN, Mexico City, Mexico; 92 Universidad Iberoamericana, Mexico City, Mexico; 93 Benemerita Universidad Autonoma de Puebla, Puebla, Mexico; 94 Universidad Autónoma de San Luis Potosí, San Luis Potosí, Mexico; 95 University of Auckland, Auckland, New Zealand; 96 University of Canterbury, Christchurch, New Zealand; 97 National Centre for Physics, Quaid-I-Azam University, Islamabad, Pakistan; 98 National Centre for Nuclear Research, Swierk, Poland; 99 Institute of Experimental Physics, Faculty of Physics, University of Warsaw, Warsaw, Poland; 100 Laboratório de Instrumentação e Física Experimental de Partículas, Lisboa, Portugal; 101 Joint Institute for Nuclear Research, Dubna, Russia; 102 Petersburg Nuclear Physics Institute, Gatchina (St. Petersburg), Russia; 103 Institute for Nuclear Research, Moscow, Russia; 104 Institute for Theoretical and Experimental Physics, Moscow, Russia; 105 Moscow Institute of Physics and Technology, Moscow, Russia; 106 National Research Nuclear University ‘Moscow Engineering Physics Institute’ (MEPhI), Moscow, Russia; 107 P.N. Lebedev Physical Institute, Moscow, Russia; 108 Skobeltsyn Institute of Nuclear Physics, Lomonosov Moscow State University, Moscow, Russia; 109 Novosibirsk State University (NSU), Novosibirsk, Russia; 110 Institute for High Energy Physics of National Research Centre ‘Kurchatov Institute’, Protvino, Russia; 111 National Research Tomsk Polytechnic University, Tomsk, Russia; 112 Faculty of Physics and Vinca Institute of Nuclear Sciences, University of Belgrade, Belgrade, Serbia; 113 Centro de Investigaciones Energéticas Medioambientales y Tecnológicas (CIEMAT), Madrid, Spain; 114 Universidad Autónoma de Madrid, Madrid, Spain; 115 Universidad de Oviedo, Oviedo, Spain; 116 Instituto de Física de Cantabria (IFCA), CSIC-Universidad de Cantabria, Santander, Spain; 117 Department of Physics, University of Ruhuna, Matara, Sri Lanka; 118 CERN, European Organization for Nuclear Research, Geneva, Switzerland; 119 Paul Scherrer Institut, Villigen, Switzerland; 120 ETH Zurich-Institute for Particle Physics and Astrophysics (IPA), Zurich, Switzerland; 121 Universität Zürich, Zurich, Switzerland; 122 National Central University, Chung-Li, Taiwan; 123 National Taiwan University (NTU), Taipei, Taiwan; 124 Department of Physics, Faculty of Science, Chulalongkorn University, Bangkok, Thailand; 125 Physics Department, Science and Art Faculty, Çukurova University, Adana, Turkey; 126 Physics Department, Middle East Technical University, Ankara, Turkey; 127 Bogazici University, Istanbul, Turkey; 128 Istanbul Technical University, Istanbul, Turkey; 129 Institute for Scintillation Materials of National Academy of Science of Ukraine, Kharkov, Ukraine; 130 National Scientific Center, Kharkov Institute of Physics and Technology, Kharkov, Ukraine; 131 University of Bristol, Bristol, UK; 132 Rutherford Appleton Laboratory, Didcot, UK; 133 Imperial College, London, UK; 134 Brunel University, Uxbridge, UK; 135 Baylor University, Waco, USA; 136 Catholic University of America, Washington DC, USA; 137 The University of Alabama, Tuscaloosa, USA; 138 Boston University, Boston, USA; 139 Brown University, Providence, USA; 140 University of California, Davis, Davis USA; 141 University of California, Los Angeles, USA; 142 University of California, Riverside, Riverside, USA; 143 University of California, San Diego, La Jolla, USA; 144 Department of Physics, University of California, Santa Barbara, Santa Barbara, USA; 145 California Institute of Technology, Pasadena, USA; 146 Carnegie Mellon University, Pittsburgh, USA; 147 University of Colorado Boulder, Boulder, USA; 148 Cornell University, Ithaca, USA; 149 Fermi National Accelerator Laboratory, Batavia, USA; 150 University of Florida, Gainesville, USA; 151 Florida International University, Miami, USA; 152 Florida State University, Tallahassee, USA; 153 Florida Institute of Technology, Melbourne, USA; 154 University of Illinois at Chicago (UIC), Chicago, USA; 155 The University of Iowa, Iowa City, USA; 156 Johns Hopkins University, Baltimore, USA; 157 The University of Kansas, Lawrence, USA; 158 Kansas State University, Manhattan, USA; 159 Lawrence Livermore National Laboratory, Livermore, USA; 160 University of Maryland, College Park, USA; 161 Massachusetts Institute of Technology, Cambridge, USA; 162 University of Minnesota, Minneapolis, USA; 163 University of Mississippi, Oxford, USA; 164 University of Nebraska-Lincoln, Lincoln, USA; 165 State University of New York at Buffalo, Buffalo, USA; 166 Northeastern University, Boston, USA; 167 Northwestern University, Evanston, USA; 168 University of Notre Dame, Notre Dame, USA; 169 The Ohio State University, Columbus, USA; 170 Princeton University, Princeton, USA; 171 University of Puerto Rico, Mayaguez, USA; 172 Purdue University, West Lafayette, USA; 173 Purdue University Northwest, Hammond, USA; 174 Rice University, Houston, USA; 175 University of Rochester, Rochester, USA; 176 Rutgers, The State University of New Jersey, Piscataway, USA; 177 University of Tennessee, Knoxville, USA; 178 Texas A& M University, College Station, USA; 179 Texas Tech University, Lubbock, USA; 180 Vanderbilt University, Nashville, USA; 181 University of Virginia, Charlottesville, USA; 182 Wayne State University, Detroit, USA; 183 University of Wisconsin-Madison, Madison, WI USA; 184 CERN, 1211 Geneva 23, Switzerland

## Abstract

A top quark mass measurement is performed using $35.9{\,\text {fb}^{-1}}$ of LHC proton–proton collision data collected with the CMS detector at $\sqrt{s}=13\,\text {TeV}$. The measurement uses the ${\mathrm {t}\overline{\mathrm {t}}}$ all-jets final state. A kinematic fit is performed to reconstruct the decay of the ${\mathrm {t}\overline{\mathrm {t}}}$  system and suppress the multijet background. Using the ideogram method, the top quark mass ($m_{\mathrm {t}}$) is determined, simultaneously constraining an additional jet energy scale factor ($\text {JSF}$). The resulting value of $m_{\mathrm {t}} =172.34\pm 0.20\,\text {(stat+JSF)} \pm 0.70\,\text {(syst)} \,\text {GeV}$ is in good agreement with previous measurements. In addition, a combined measurement that uses the ${\mathrm {t}\overline{\mathrm {t}}}$ lepton+jets and all-jets final states is presented, using the same mass extraction method, and provides an $m_{\mathrm {t}}$ measurement of $172.26\pm 0.07\,\text {(stat+JSF)} \pm 0.61\,\text {(syst)} \,\text {GeV}$. This is the first combined $m_{\mathrm {t}}$ extraction from the lepton+jets and all-jets channels through a single likelihood function.

## Introduction

The top quark [1, 2] is the most massive known fundamental particle and its mass $m_{\mathrm {t}}$ is an important parameter of the standard model (SM) of particle physics. Precise measurements of $m_{\mathrm {t}}$ can be used to test the internal consistency of the SM [3–5] and to search for new physical phenomena. Since the top quark dominates the higher-order corrections to the Higgs boson mass, a precise $m_{\mathrm {t}}$ determination is crucial to put constraints on the stability of the electroweak vacuum [6, 7].

At the CERN LHC, top quarks are predominantly produced in quark-antiquark pairs (${\mathrm {t}\overline{\mathrm {t}}}$) through the gluon fusion process, and decay almost exclusively to a bottom quark and a $\mathrm {W}$ boson. Each ${\mathrm {t}\overline{\mathrm {t}}}$ event can be classified through the decays of the $\mathrm {W}$ bosons. Events in the all-jets final state correspond to those that have both $\mathrm {W}$ bosons decaying further into $\mathrm {q}\mathrm {\overline{q}}'$ pairs, while events in the lepton+jets final state have one $\mathrm {W}$ boson decaying to a charged lepton and a neutrino.

This paper presents a measurement of $m_{\mathrm {t}}$ obtained in the ${\mathrm {t}\overline{\mathrm {t}}}$ all-jets decay channel using proton–proton ($\mathrm {p}\mathrm {p}$) collision data taken in 2016 by the CMS experiment at a center-of-mass energy of $\sqrt{s}=13\,\text {TeV}$, corresponding to an integrated luminosity of $35.9{\,\text {fb}^{-1}}$. The two bottom quarks and the four light quarks from the ${\mathrm {t}\overline{\mathrm {t}}}$ decay are all required to be physically separated in the laboratory frame of reference, and the nominal experimental signature is therefore characterized by six jets in the detector.

Although this final state provides the largest branching fraction of all ${\mathrm {t}\overline{\mathrm {t}}}$  decays, this measurement of $m_{\mathrm {t}}$ is particularly challenging, because of the large background from multijet production. A kinematic fit of the decay products to the ${\mathrm {t}\overline{\mathrm {t}}}$ hypothesis is therefore employed to separate signal from background events.

The value of $m_{\mathrm {t}}$ is extracted using the ideogram method [8, 9], which is based on a likelihood function that depends either just on the mass parameter $m_{\mathrm {t}}$, or on $m_{\mathrm {t}}$ combined with an additional jet energy scale factor (JSF). In the second case, the invariant mass of the two jets associated with the $\mathrm {W}\rightarrow \mathrm {q}\overline{\mathrm {q}}'$ decay serves as an observable to directly estimate the JSF.

Previous measurements in this decay channel have been performed by Tevatron and LHC experiments at lower center-of-mass energies [10–14]. The most precise one of these has been obtained by CMS at $\sqrt{s}=8\,\text {TeV}$, resulting in a mass of $m_{\mathrm {t}} =172.32\pm 0.25\,\text {(stat+JSF)} \pm 0.59\,\text {(syst)} \,\text {GeV}$. Combining the results of several measurements using different final states at $\sqrt{s}=7$ and $8\,\text {TeV}$, ATLAS and CMS reported values of $m_{\mathrm {t}} =172.69\pm 0.48\,\text {GeV}$ [15] and $172.44\pm 0.48\,\text {GeV}$ [12], respectively, while a value of $m_{\mathrm {t}} =174.30\pm 0.65\,\text {GeV}$ was obtained by combining the Tevatron results [16].

The top quark mass has been measured for the first time with $\mathrm {p}\mathrm {p}$ data at $\sqrt{s} = 13\,\text {TeV}$, using the lepton+jets channel [17], yielding a value of $m_{\mathrm {t}} = 172.25\pm 0.08\,\text {(stat+JSF)} \pm 0.62\,\text {(syst)} \,\text {GeV}$. A measurement using both ${\mathrm {t}\overline{\mathrm {t}}}$ all-jets and lepton+jets events is presented here. This is possible since the two measurements use the same mass extraction method, so a single likelihood can be used, rather than just combining the two results statistically. With this approach, no assumptions on correlations between different uncertainties of the measurements have to be made. This is the first report of a combined $m_{\mathrm {t}}$ measurement in the lepton+jets and all-jets final states using a single likelihood function.

## The CMS detector and event reconstruction

The central feature of the CMS apparatus is a superconducting solenoid of 6 $\text { m}$ internal diameter, providing a magnetic field of 3.8 $\text { T}$. Within the solenoid volume are a silicon pixel and strip tracker, a lead tungstate crystal electromagnetic calorimeter (ECAL), and a brass and scintillator hadron calorimeter (HCAL), each composed of a barrel and two endcap sections. Forward calorimeters extend the pseudorapidity ($\eta$) coverage provided by the barrel and endcap detectors. Muons are detected in gas-ionization chambers embedded in the steel flux-return yoke outside the solenoid.

Events of interest are selected using a two-tiered trigger system [18]. The first level, composed of custom hardware processors, uses information from the calorimeters and muon detectors to select events within a time interval of 4$\,\mu \text {s}$ , resulting in a trigger rate of around 100 $\text { kHz}$. The second level, known as the high-level trigger (HLT), consists of a farm of processors running a version of the full event reconstruction software optimized for fast processing, and reduces the event rate to around 1 $\text { kHz}$ before data storage.

The particle-flow (PF) algorithm [19] aims to reconstruct and identify each individual particle in an event, with an optimized combination of information from the various elements of the CMS detector. The energy of photons is obtained from the ECAL measurement. The energy of electrons is determined from a combination of the electron momentum at the primary interaction vertex as determined by the tracker, the energy of the corresponding ECAL cluster, and the energy sum of all bremsstrahlung photons spatially compatible with originating from the electron track. The energy of muons is obtained from the curvature of the corresponding track. The energy of charged hadrons is determined from a combination of their momentum measured in the tracker and the matching ECAL and HCAL energy deposits, corrected for zero-suppression effects and for the response function of the calorimeters to hadronic showers. Finally, the energy of neutral hadrons is obtained from the corresponding corrected ECAL and HCAL energy.

The reconstructed vertex with the largest value of summed physics-object $p_{\mathrm {T}} ^2$ is taken to be the primary proton–proton interaction vertex. The physics objects are the jets, clustered using the jet finding algorithm [20, 21] with the tracks assigned to the vertex as inputs, and the associated missing transverse momentum, taken as the negative vector sum of the transverse momentum $p_{\mathrm {T}}$ of those jets.

Jets are clustered from PF objects using the anti-$k_{\mathrm {T}}$ algorithm with a distance parameter of 0.4 [20–22]. Jet momentum is determined as the vectorial sum of all particle momenta in the jet, and is found from simulation to be within 5–10% of the true momentum over the whole $p_{\mathrm {T}}$ spectrum and detector acceptance. Additional proton–proton interactions within the same or nearby bunch crossings (pileup) can contribute additional tracks and calorimetric energy depositions to the jet momentum. To mitigate this effect, tracks identified to be originating from pileup vertices are discarded, and an offset correction is applied to correct for remaining contributions from neutral hadrons. Jet energy corrections (JECs) are derived from simulation to bring the measured response of jets to that of particle level jets on average. In situ measurements of the momentum balance in dijet, photon+jet, $\mathrm {Z}$ +jet, and multijet events are used to account for any residual differences in the jet energy scale in data and simulation [23]. Additional selection criteria are applied to each jet to remove jets dominated by anomalous contributions from various subdetector components or reconstruction failures [24].

A more detailed description of the CMS detector, together with a definition of the coordinate system used and the relevant kinematic variables, can be found in Ref. [25].

## Event selection and simulation

Only jets with $p_{\mathrm {T}} >30\,\text {GeV}$ reconstructed within $|\eta | < 2.4$ are used in the analysis. For the identification of jets originating from the hadronization of $\mathrm {b}$ quarks, the combined secondary vertex algorithm (CSVv2) $\mathrm {b}$ tagger is used [26]. The chosen working point provides an identification efficiency of approximately $50\%$ with a probability of misidentifying a $\mathrm {u}$/$\mathrm {d}$/$\mathrm {s}$ quark jet or gluon jet as being a bottom jet of approximately $0.1\%$, and a misidentification probability for $\mathrm {c}$ quark jets of $2\%$. The hadronic activity, used for the event selection, is defined as the scalar $p_{\mathrm {T}}$ sum of all jets in the event,

$$\begin{aligned} H_{\mathrm {T}} \equiv \sum _{\text {jets}}p_{\mathrm {T}}. \end{aligned}$$

Data events are selected using an HLT that requires the presence of at least six PF jets with $p_{\mathrm {T}} >40\,\text {GeV}$ and $H_{\mathrm {T}} >450\,\text {GeV}$. Additionally, the HLT requires at least one jet to be $\mathrm {b}$ tagged.

In the offline selection, an event must contain a well reconstructed vertex localized within 24 cm in the *z* direction and 2$\,\text {cm}$ in the *x*–*y* plane around the nominal interaction point. Selected events are required to contain at least six jets, at least two of which have to be tagged as $\mathrm {b}$ jets. The sixth jet ($\text {jet}_6$), ordered in decreasing $p_{\mathrm {T}}$, must fulfill $p_{\mathrm {T}} (\text {jet}_6) > 40\,\text {GeV}$, and $H_{\mathrm {T}} >450\,\text {GeV}$ is required. The two $\mathrm {b}$ jets must be separated in $\varDelta R = \sqrt{\smash [b]{\varDelta \phi ^2 + \varDelta \eta ^2}}$ by $\varDelta R(\mathrm {b}\overline{\mathrm {b}}) > 2.0$.

The ${\mathrm {t}\overline{\mathrm {t}}}$ signal is simulated at an $m_{\mathrm {t}}$ of $172.5\,\text {GeV}$ using the powheg  v2 [27–29] matrix-element (ME) generator in next-to-leading order (NLO) perturbative quantum chromodynamics (QCD). For the parton distribution functions (PDFs), the NNPDF3.0 NLO set [30] is used with the strong coupling constant value of $\alpha _S =0.118$. This is one of the first PDF sets to include the total ${\mathrm {t}\overline{\mathrm {t}}}$ cross section measurements from ATLAS and CMS at $\sqrt{s}=7$ and $8\,\text {TeV}$ as input. The parton shower (PS) and hadronization are handled by pythia  8.219 [31] using the CUETP8M2T4 tune [32, 33] and Geant4 is used to simulate the response of the CMS detector [34]. The simulated signal sample is normalized to the integrated luminosity of the data sample using a cross section of $\sigma _{{\mathrm {t}\overline{\mathrm {t}}}} = 832\,\text { pb}$, calculated at next-to-next-to-leading order in QCD including resummation of next-to-next-to-leading logarithmic soft gluon terms [35]. In addition to the default sample, six other samples are used assuming top quark masses of 166.5, 169.5, 171.5, 173.5, 175.5, and 178.5$\,\text {GeV}$, and using the corresponding cross sections.

For simulated events, a trigger emulation is used. The residual differences in the trigger efficiency between data and simulation are corrected by applying scale factors to the simulated events. These are obtained by measuring the trigger efficiency with respect to a reference $H_{\mathrm {T}}$  trigger for both data and simulation. The parameterized ratio as a function of $p_{\mathrm {T}} (\text {jet}_6)$ and $H_{\mathrm {T}}$ is used to reweight the simulated events. Additional $\mathrm {p}\mathrm {p}$ collisions are included in the simulated events. These are weighted to match the pileup distribution in data. Finally, corrections to the jet energy scale and resolution, as well as to the $\mathrm {b}$ tagging efficiency and misidentification rate, are applied to the simulated events.

## Kinematic fit and background estimation

To improve the resolution of the top quark mass and decrease the background contribution, a kinematic fit is applied. It exploits the known topology of the signal events, i.e., pair production of a heavy particle and antiparticle, each decaying to $\mathrm {W}\mathrm {b}$ with $\mathrm {W}\rightarrow \mathrm {q}\overline{\mathrm {q}}'$. The three-momenta of the jets are fitted such that

$$\begin{aligned} {\chi ^2}&= \sum _{j\in \text {jets}}\left[ \frac{\left( {p_{\mathrm {T}}}_j^\text {reco} - {p_{\mathrm {T}}}_j^\text {fit}\right) ^2}{\sigma _{{p_{\mathrm {T}}}_j}^2} + \frac{\left( \eta _j^\text {reco} - \eta _j^\text {fit}\right) ^2}{\sigma _{\eta _j}^2}\right. \\&\qquad \left. +\frac{\left( \phi _j^\text {reco} - \phi _j^\text {fit}\right) ^2}{\sigma _{\phi _j}^2} \right] \end{aligned}$$

is minimized, where all jets assigned to the ${\mathrm {t}\overline{\mathrm {t}}}$ decay system are considered. The labels “reco” and “fit” denote the components of the originally reconstructed and the fitted jets, respectively, and the corresponding resolutions are labeled $\sigma _X$. The minimization is performed, constraining the invariant mass of the jets assigned to each $\mathrm {W}$ boson decay to $m_{\mathrm {W}}=80.4\,\text {GeV}$. As an additional constraint, the two top quark candidates are required to have equal invariant masses.

All possible parton-jet assignments are tested using the leading six jets in the event, but only $\mathrm {b}$-tagged jets are used as $\mathrm {b}$ candidates and equivalent choices (e.g., swapping the two jets originating from one $\mathrm {W}$ boson) are not considered separately. Of the remaining 12 possibilities, only the assignment yielding the smallest ${\chi ^2}$ is used in the following. The ${\chi ^2}$ value can be used as a goodness-of-fit (gof) measure. For three degrees of freedom, it is translated into a *p*-value of

$$\begin{aligned} P_\text {gof} \equiv 1-{\text {erf}} \left( \sqrt{\frac{{\chi ^2}}{2}}\right) + \sqrt{\frac{2{\chi ^2}}{\pi }}\mathrm {e}^{-{\chi ^2}/2}. \end{aligned}$$

Events are required to fulfill $P_\text {gof} >0.1$ for the best assignment.

In simulation, event generator information can be used to validate the assignment of the reconstructed jets to the top quark decay products. Events are classified accordingly as *correct* or *wrong* permutations. A parton-jet assignment is considered correct if the jets can be matched unambiguously to the right partons within $\varDelta R < 0.3$. Wrong permutations can occur because of a wrong parton-jet assignment, yielding the smallest ${\chi ^2}$ or jets being out of acceptance, not being reconstructed, or failing the identification requirements.

**Fig. 1:**
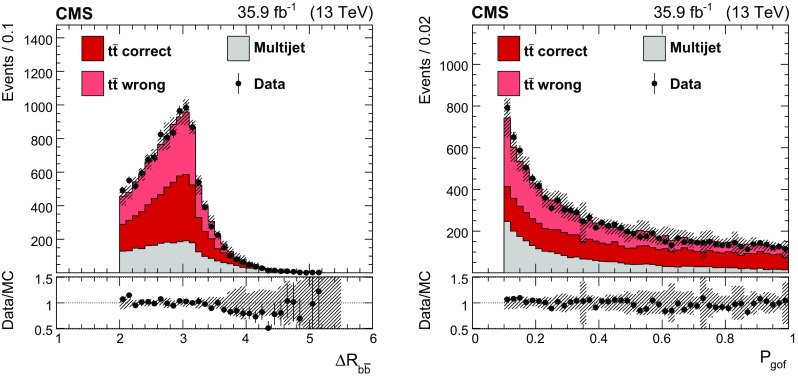
The $\varDelta R(\mathrm {b}\overline{\mathrm {b}})$ (left) and $P_\text {gof}$ (right) distributions of data compared to simulated signal and the multijet background estimate. For each event, the parton-jet assignment yielding the smallest $\chi ^2$ in the kinematic fit is used. The simulated signal events are classified as correct or wrong assignments and displayed separately, and the distributions are normalized to the integrated luminosity. For the background estimate, the total normalization is given by the difference of observed data events and expected signal events. The hashed bands represent the total uncertainty in the complete prediction. The lower panels show the ratio of data and prediction

The $P_\text {gof}$ distribution is displayed in Fig. 1 (right). Requiring $P_\text {gof} >0.1$ increases the fraction of correct permutations from 6 to $51\%$. The fitted top quark mass ($m_{\mathrm {t}}^\text {fit}$) is calculated as the invariant mass of the corresponding jets returned by the kinematic fit. Compared to the mass calculated from the originally reconstructed jets, the mass resolution is improved from 14.0 to $8.8\,\text {GeV}$ for the correct parton-jet assignments, where, in both cases, the same events passing the $P_\text {gof} >0.1$ requirement are used.

The $\varDelta R(\mathrm {b}\overline{\mathrm {b}}) > 2.0$ and $P_\text {gof} >0.1$ requirements greatly reduce the background from QCD multijet production from approximately 80 to 25%, but a significant number of multijet events enters the signal selection owing to the large production cross section of that background contribution. These events can fulfill the goodness-of-fit criterion because of combinatorial chance, but not because of an underlying decay topology. Therefore, it is assumed that $\mathrm {b}$ jets can be exchanged with light-flavor jets for the estimation of the background from data, because the probability for mimicking the ${\mathrm {t}\overline{\mathrm {t}}}$ topology is the same.

For the background estimation, the same selection as for the signal is applied, as described above, but instead of requiring two $\mathrm {b}$-tagged jets, events with exactly zero $\mathrm {b}$-tagged jets are used. For this veto, a very loose working point is used for the $\mathrm {b}$ tagger, to suppress contamination from ${\mathrm {t}\overline{\mathrm {t}}}$  events in this QCD-enriched sample. A prescaled trigger similar to the signal trigger is used for this selection, which does not require the presence of $\mathrm {b}$ jets. The kinematic fit is applied as before, but here any of the six light-flavor jets can be assigned to the partons originating from the $\mathrm {W}$ decays, as well as to the partons serving as $\mathrm {b}$ quarks, leading to 90 possible permutations that have to be evaluated. This method allows one to determine the kinematic distributions of the background, but the normalization is unknown. In all plots, the background is normalized to the difference of the number of data events and the number of expected signal events. This data sample contains approximately five times the number of expected background events, so it provides good statistical precision.

**Fig. 2:**
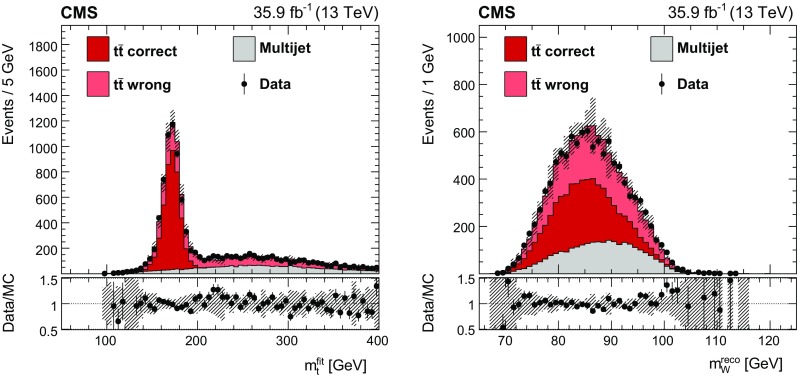
The fitted top quark mass (left) and reconstructed $\mathrm {W}$ boson mass (right) distributions of data compared to simulated signal and the multijet background estimate. The shown reconstructed $\mathrm {W}$ boson mass is the average mass of the two W bosons in the event. For each event, the parton-jet assignment yielding the smallest $\chi ^2$ in the kinematic fit is used. The simulated signal events are classified as correct or wrong assignments and displayed separately, and the distributions are normalized to the integrated luminosity. For the background estimate, the total normalization is given by the difference of observed data events and expected signal events. The hashed bands represent the total uncertainty in the prediction. The lower panels show the ratio of data and prediction

The final selected data set consists of 10,799 events with a signal purity of $75\%$. Figure 1 shows the distributions of the separation of the two $\mathrm {b}$ jets $\varDelta R(\mathrm {b}\overline{\mathrm {b}})$ and the quantity $P_\text {gof}$ in data, compared to the background estimate and ${\mathrm {t}\overline{\mathrm {t}}}$ simulation. For the ${\mathrm {t}\overline{\mathrm {t}}}$  signal, correct and wrong parton-jet assignments are shown separately. The corresponding distributions of $m_{\mathrm {t}}^\text {fit}$ and the reconstructed $\mathrm {W}$ boson mass $m_{\mathrm {W}}^\text {reco}$, calculated from the originally reconstructed jets, are shown in Fig. 2. These two quantities are used in the top quark mass extraction described in the following section.

## Ideogram method

For the extraction of $m_{\mathrm {t}}$, the ideogram method is used [8, 9]. Simultaneously, a JSF is determined that is used in addition to the standard CMS jet energy calibration [12] to reduce the corresponding systematic uncertainty. The distributions of $m_{\mathrm {t}}^\text {fit}$ obtained from the kinematic fit and $m_{\mathrm {W}}^\text {reco}$ are used in a combined fit. For $m_{\mathrm {W}}^\text {reco}$, the average mass of the two $\mathrm {W}$ bosons in an event is used.

The likelihood

$$\begin{aligned} \mathcal {L}\left( m_{\mathrm {t}},\text {JSF}\right)&= P\left( \text {sample}|m_{\mathrm {t}},\text {JSF}\right) \\&= \prod _{\text {events}}P\left( \text {event}|m_{\mathrm {t}},\text {JSF}\right) \\&= \prod _{\text {events}}P\left( m_{\mathrm {t}}^\text {fit},m_{\mathrm {W}}^\text {reco} |m_{\mathrm {t}},\text {JSF}\right) \end{aligned}$$

is maximized, yielding the best fit values for $m_{\mathrm {t}}$ and JSF. A prior probability for the $\text {JSF}$ can be incorporated by maximizing

$$\begin{aligned} P(\text {JSF}) P\left( \text {sample}|m_{\mathrm {t}},\text {JSF}\right) \end{aligned}$$

instead. Treating $m_{\mathrm {t}}^\text {fit}$ and $m_{\mathrm {W}}^\text {reco}$ as uncorrelated, as verified using simulated events, the probability $P\left( m_{\mathrm {t}}^\text {fit},m_{\mathrm {W}}^\text {reco} |m_{\mathrm {t}},\text {JSF} \right)$ factorizes into

$$\begin{aligned}&P\left( m_{\mathrm {t}}^\text {fit},m_{\mathrm {W}}^\text {reco} |m_{\mathrm {t}},\text {JSF}\right) \\&\quad {}={}f_\text {sig} P\left( m_{\mathrm {t}}^\text {fit},m_{\mathrm {W}}^\text {reco} |m_{\mathrm {t}},\text {JSF}\right) \\&\qquad {}+{}\left( 1-f_\text {sig}\right) P_\text {bkg}\left( m_{\mathrm {t}}^\text {fit},m_{\mathrm {W}}^\text {reco} \right) \\&\quad {}={}f_\text {sig} \sum _{j}f_{j}P_{j}\left( m_{\mathrm {t}}^\text {fit} |m_{\mathrm {t}},\text {JSF}\right) P_{j}\left( m_{\mathrm {W}}^\text {reco} |m_{\mathrm {t}},\text {JSF}\right) \\&\qquad {}+{}\left( 1-f_\text {sig}\right) P_\text {bkg}\left( m_{\mathrm {t}}^\text {fit} \right) P_\text {bkg}\left( m_{\mathrm {W}}^\text {reco} \right) , \end{aligned}$$

where $f_j$ with $j\in \left\{ \text {correct}, \text {wrong}\right\}$ is the relative fraction of the different permutation cases and $f_\text {sig}$ is the signal fraction.

The probability densities $P_{j}\left( m_{\mathrm {t}}^\text {fit} |m_{\mathrm {t}},\text {JSF}\right)$ and $P_{j}\left( m_{\mathrm {W}}^\text {reco} |m_{\mathrm {t}},\text {JSF}\right)$ for the signal are described by analytic functions parametrized in $m_{\mathrm {t}}$ and JSF. For the determination of the parameters, a simultaneous fit to simulated samples for seven different generated top quark masses $m_{\mathrm {t}}^\text {gen}$ and five different input JSF values is used. The background shape is described by a spline interpolation as a function of $m_{\mathrm {t}}^\text {fit}$ and $m_{\mathrm {W}}^\text {reco}$, but independent of the model parameters $m_{\mathrm {t}}$ and JSF.

Three variations of a maximum likelihood fit are performed to extract the top quark mass. In the one-dimensional (1D) analysis, the JSF is fixed to unity (corresponding to a Dirac delta function for the prior probability), i.e., the standard CMS jet energy calibration. For the two-dimensional (2D) analysis, the JSF is a free parameter in the maximum likelihood fit, making possible a compensation of part of the systematic uncertainties. The signal fraction and correct permutation fraction are free parameters in both cases. The third (hybrid) method is a weighted combination of both approaches, corresponding to a measurement with a Gaussian constraint on the JSF around unity. In the limit of an infinitely narrow JSF constraint, the hybrid method is identical to the 1D method, while for an infinitely broad prior probability distribution, the 2D method is recovered. The width of the Gaussian constraint in the hybrid method is optimized to yield the smallest total uncertainty.

**Fig. 3:**
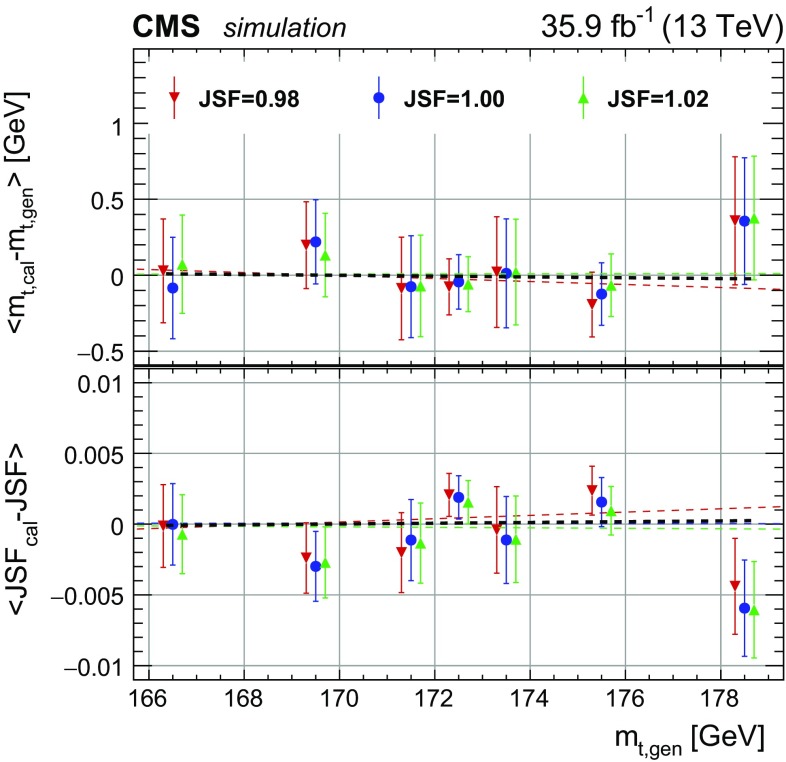
Difference between extracted and generated top quark masses (upper panel) and JSFs (lower panel) for different input masses and JSFs after the calibration in the all-jets channel. The values are extracted using the 2D method

To calibrate the mass extraction method, pseudo-experiments are performed for the seven different generated values of $m_{\mathrm {t}}^\text {gen}$ and three input JSF values (0.98, 1.00, and 1.02). The extracted $m_{\mathrm {t}}$ and JSF values are compared to the input values and the residual slopes in $m_{\mathrm {t}}^\text {gen}$ and JSF are used as calibration. The residual biases after the calibration are shown in Fig. 3 for pseudo-experiments with different JSF and $m_{\mathrm {t}}^\text {gen}$ values. As expected, neither a significant residual offset nor a slope are observed after the calibration procedure.

## Systematic uncertainties

A summary of the systematic uncertainty sources is shown in Table 1. The corresponding values are obtained from pseudo-experiments, using Monte Carlo (MC) signal samples with variations of the individual systematic uncertainty sources. In the following, details for the determination of the most important uncertainties are given. Most systematic uncertainty sources are shifted by $\pm 1$ standard deviation, and the absolute value of the largest resulting shifts in $m_{\mathrm {t}}$ and JSF are quoted as systematic uncertainties for the measurement. For some uncertainties, different models are compared, and are described individually. The maximum of the statistical uncertainty on the observed shift and the shift itself is used as the systematic uncertainty.

**Table 1:** List of systematic uncertainties for the all-jets channel. The signs of the shifts ($\delta x = x_\text {variation} - x_\text {nominal}$) correspond to the $+\,1$ standard deviation variation of the systematic uncertainty source. For linear sums of the uncertainty groups, the relative signs have been considered. Shifts determined using dedicated samples for the systematic variation are displayed with the corresponding statistical uncertainty

	2D		1D	Hybrid	
	$\delta m_{\mathrm {t}} ^{\text {2D}}$	$\delta \text {JSF} ^{\text {2D}}$	$\delta m_{\mathrm {t}} ^{\text {1D}}$	$\delta m_{\mathrm {t}} ^{\text {hyb}}$	$\delta \text {JSF} ^{\text {hyb}}$
	[GeV]	[%]	[GeV]	[GeV]	[%]
*Experimental uncertainties*					
Method calibration	0.06	0.2	0.06	0.06	0.2
JEC (quad. sum)	0.18	0.3	0.73	0.15	0.2
Intercalibration	$-$ 0.04	$-$ 0.1	$+$ 0.12	$-$ 0.04	$-$ 0.1
MPFInSitu	$-$ 0.03	0.0	$+$ 0.22	$+$ 0.08	$+$ 0.1
Uncorrelated	$-$ 0.17	$-$ 0.3	$+$ 0.69	$+$ 0.12	$+$ 0.2
Jet energy resolution	$-$ 0.09	$+$ 0.2	$+$ 0.09	$-$ 0.04	$+$ 0.1
$\mathrm {b}$ tagging	0.02	0.0	0.01	0.02	0.0
Pileup	$-$ 0.06	$+$ 0.1	0.00	$-$ 0.04	$+$ 0.1
Background	0.10	0.1	0.03	0.07	0.1
Trigger	$+$ 0.04	$-$ 0.1	$-$ 0.04	$+$ 0.02	$-$ 0.1
*Modeling uncertainties*					
JEC flavor (linear sum)	$-$ 0.35	$+$ 0.1	$-$ 0.31	$-$ 0.34	0.0
Light quarks (uds)	$+$ 0.10	$-$ 0.1	$-$ 0.01	$+$ 0.07	$-$ 0.1
Charm	$+$ 0.03	0.0	$-$ 0.01	$+$ 0.02	0.0
Bottom	$-$ 0.29	0.0	$-$ 0.29	$-$ 0.29	0.0
Gluon	$-$ 0.19	$+$ 0.2	$+$ 0.03	$-$ 0.13	$+$ 0.2
$\mathrm {b}$ jet modeling (quad. sum)	0.09	0.0	0.09	0.09	0.0
$\mathrm {b}$ frag. Bowler–Lund	$-$ 0.07	0.0	$-$ 0.07	$-$ 0.07	0.0
$\mathrm {b}$ frag. Peterson	$-$ 0.05	0.0	$-$ 0.04	$-$ 0.05	0.0
Semileptonic $\mathrm {b}$ hadron decays	$-$ 0.03	0.0	$-$ 0.03	$-$ 0.03	0.0
PDF	0.01	0.0	0.01	0.01	0.0
Ren. and fact. scales	0.05	0.0	0.04	0.04	0.0
ME/PS matching	$+\,0.32\pm 0.20$	$-$ 0.3	$-\,0.05\pm \,0.14$	$+\,0.24\pm 0.18$	$-$ 0.2
ISR PS scale	$+\,0.17\pm 0.17$	$-$ 0.2	$+\,0.13\pm \,0.12$	$+\,0.12\pm 0.14$	$-$ 0.1
FSR PS scale	$+\,0.22\pm \,0.12$	$-$ 0.2	$+\,0.11\pm \,0.08$	$+\,0.18\pm 0.11$	$-$ 0.1
Top quark $p_{\mathrm {T}}$	$+$ 0.03	0.0	$+$ 0.02	$+$ 0.03	0.0
Underlying event	$+\,0.16\pm \,0.19$	$-$ 0.3	$-\,0.07\pm 0.14$	$+\,0.10\pm 0.17$	$-$ 0.2
Early resonance decays	$+\,0.02\pm \,0.28$	+ 0.4	$+\,0.38\pm 0.19$	$+\,0.13\pm \,0.24$	+ 0.3
CR modeling (max. shift)	$+\,0.41\pm \,0.29$	$-$ 0.4	$-\,0.43\pm 0.20$	$-\,0.36\pm 0.25$	$-$ 0.3
“gluon move” (ERD on)	$+\,0.41\pm \,0.29$	$-$ 0.4	$+\,0.10\pm \,0.20$	$+\,0.32\pm 0.25$	$-$ 0.3
“QCD inspired” (ERD on)	$-\,0.32\pm \,0.29$	$-$ 0.1	$-\,0.43\pm \,0.20$	$-\,0.36\pm 0.25$	$-$ 0.1
Total systematic	0.81	0.9	1.03	0.70	0.7
Statistical (expected)	0.21	0.2	0.16	0.20	0.1
Total (expected)	0.83	0.9	1.04	0.72	0.7

- *Method calibration* The quadratic sum of the statistical uncertainty and the residual bias of the calibration curve (shown in Fig. 3) after the calibration is used as the systematic uncertainty.
- *JECs* Jet energies are scaled up and down according to the $p_{\mathrm {T}}$- and $\eta$-dependent data/simulation uncertainties [23]. The correlation groups (called Intercalibration, MPFInSitu, and Uncorrelated) follow the recommendations documented in Ref. [36].
- *Jet energy resolution* Since the jet energy resolution measured in data is worse than in simulation, the simulation is modified to correct for the difference [23]. The jet energy resolution in the simulation is varied up and down within the uncertainty.
- $\mathrm {b}$ *tagging* The $p_{\mathrm {T}}$-dependent uncertainty of the $\mathrm {b}$ tagging efficiencies and misidentification rates of the CSVv2 $\mathrm {b}$ tagger [26] are taken into account by reweighting the simulated events accordingly.
- *Pileup* To estimate the uncertainty in the determination of the number of pileup events and the reweighting procedure, the inelastic proton–proton cross section [37] used in the determination is varied by ${\pm }\,4.6\%$.
- *Background* An uncertainty in the background prediction is obtained by applying the method to simulation and comparing the obtained estimate to the direct simulation, i.e., generated QCD multijet events passing the signal selection. A linear fit to the ratio is consistent with a constant value of unity. The slope is varied up and down within its uncertainty and used to reweight the events used for the determination of the background probability density function.
- *Trigger* To estimate the uncertainty in the trigger selection, the data/simulation scale factor described in Sect. 3 is omitted. Additionally, a base trigger requiring the presence of one muon is used to obtain the correction factor. The maximum of the observed shifts with respect to the nominal correction is quoted as an uncertainty.
- *JEC flavor* The difference between Lund string fragmentation and cluster fragmentation is evaluated comparing pythia  6.422 [38] and herwig++  2.4 [39]. The jet energy response is compared separately for each jet flavor [23]. Uncertainties for jets from different quark flavors and gluons are added linearly, which takes into account possible differences between the measured JSF, which is mainly sensitive to light quarks and gluons, and the $\mathrm {b}$ jet energy scale.
- $\mathrm {b}$ *jet modeling* The uncertainty associated with the fragmentation of $\mathrm {b}$ quarks is split into three components. The Bowler–Lund fragmentation function is varied within its uncertainties as determined by the ALEPH and DELPHI Collaborations [40, 41]. As an alternative model of the fragmentation into $\mathrm {b}$ hadrons, the Peterson fragmentation function is used and the difference obtained relative to the Bowler–Lund fragmentation function is assigned as an uncertainty. The third uncertainty source taken into account is the semileptonic $\mathrm {b}$ hadron branching fraction, which is varied by $-0.45\%$ and $+\,0.77\%$, motivated by measurements of ${\mathrm {B}^0}$/${\mathrm {B}^{+}}$decays and their corresponding uncertainties [42].
- *PDF* The 100 PDF replicas of the NNPDF3.0 NLO ($\alpha _S = 0.118)$ set are used to repeat the analysis [30]. The variance of the results is used to determine the PDF uncertainty. In addition, the $\alpha _S$ value is changed to 0.117 and 0.119. The maximum of the PDF uncertainty and the $\alpha _S$ variations is quoted as uncertainty.
- *Renormalization and factorization scales* The renormalization and factorization scales for the ME calculation are varied. Both are multiplied independently from each other, and simultaneously by factors of 0.5 and 2 with respect to the default values. This is achieved by appropriately reweighting simulated events. The quoted uncertainty corresponds to the envelope of the resulting shifts.
- *ME/PS matching* The matching of the powheg ME calculations to the pythia PS is varied by shifting the parameter $h_{\text {damp}}=1.58^{+\,0.66}_{-0.59}$ [33] within the uncertainties. The jet response $p_{\mathrm {T}} ^{\text {reco}}/p_{\mathrm {T}} ^{\text {gen}}$ as a function of $p_{\mathrm {T}} ^{\text {gen}}$ is rescaled in the variation samples to reproduce the response observed in the default sample.
- *ISR PS scale* For initial-state radiation (ISR), the PS scale is varied in pythia. The ISR PS scale is multiplied by factors of 2 and 0.5 in dedicated MC samples.
- *FSR PS scale* The PS scale used for final-state radiation (FSR) is scaled up by $\sqrt{2}$ and down by $1/\sqrt{2}$ [32], affecting the fragmentation and hadronization, as well additional jet emission. The jet response is rescaled in the variation samples to reproduce the response observed in the default sample.
- *Top quark* $p_{\mathrm {T}}$ Recent calculations suggest that the top quark $p_{\mathrm {T}}$ spectrum is strongly affected by next-to-next-to-leading-order effects [43]. The $p_{\mathrm {T}}$ of the top quark in simulation is varied to match the distribution measured by CMS [44, 45] and its impact on the $m_{\mathrm {t}}$ measurement is quoted as a systematic uncertainty.
- *Underlying event* Measurements of the underlying event have been used to tune pythia parameters describing nonperturbative QCD effects [32, 33]. The parameters of the tune are varied within their uncertainties.
- *Early resonance decays* Modeling of color reconnection (CR) introduces systematic uncertainties which are estimated by comparing different CR models and settings. In the default sample, the top quark decay products are not included in the CR process. This setting is compared to the case of including the decay products by enabling early resonance decays (ERD) in pythia 8.
- *CR modeling* In addition to the default model used in pythia 8, two alternative CR models are used, namely a model with string formation beyond leading color (“QCD inspired”) [46] and a model allowing the gluons to be moved to another string (“gluon move”) [47]. Underlying event measurements are used to tune the parameters of all models [32, 33]. The largest shifts induced by the variations are assigned as the CR uncertainty.
  This approach, as well as the ERD variation, is new relative to the Run 1 results at $\sqrt{s}=7$ and $8\,\text {TeV}$, because these CR models have become only recently available in pythia 8. The new models were first used to evaluate the $m_{\mathrm {t}}$ uncertainty due to CR in Ref. [17]. Like in this analysis, the same increase in systematic uncertainty with respect to the Run 1 result has been observed.

A summary of the systematic uncertainties described above is given in Table 1. In Ref. [17], an ME generator uncertainty has been considered: Instead of using powheg  v2 as ME generator, the MadGraph 5_amc@nlo 2.2.2 generator with the FxFx matching scheme is used [48, 49]. The difference between the results obtained with the two generators is $\delta m_{\mathrm {t}} ^{\text {hyb}}=+\,0.31\pm 0.52$ for the hybrid method in the all-jets channel. However, this is not significant because of the insufficient statistical precision of the available MadGraph 5_amc@nlo sample. Since the radiation after the top quark decay is described by pythia, no significant impact of the ME generator choice is expected beyond the variation of the PS scales and matching. Therefore, no ME generator uncertainty is considered in the total uncertainty of the measurement, but the number is just quoted here as a cross-check.

## Results

For the 2D fit using the 10 799 ${\text{t}}{\bar{\text{t}}}$ all-jets candidate events, the extracted parameters are

$$\begin{aligned} m_{\mathrm {t}} ^{\text {2D}}&= 172.43\pm 0.22 {\text{ (stat+JSF)}} \pm 0.81 {\text { (syst) GeV and}} \\ {\text {JSF}}^{\text{2D}}&= 0.996\pm 0.002 {\text{ (stat)}} \pm 0.009 {\text {(syst)}}. \end{aligned}$$

The corresponding 1D and hybrid fits yield instead

$$\begin{aligned} m_{\mathrm {t}} ^{\text {1D}}&= 172.13\pm 0.17\text { (stat)} \pm 1.03 {\text{ (syst) GeV}},\\ m_{\mathrm {t}} ^{\text {hyb}}&= 172.34\pm 0.20 {\text { (stat+JSF)}} \pm 0.70\,{\text {(syst) GeV, and}}\\ {\text {JSF}}^{\text {hyb}}&= 0.997\pm 0.002 {\text { (stat)}} \pm 0.007\,{\text {(syst)}}. \end{aligned}$$

In all cases the fitted values for the fraction of correct assignments, as well as the background fraction, are in agreement with the values expected from simulation. The hybrid measurement of $172.34\pm 0.20\,\text {(stat+JSF)} \pm 0.43\,\text {(CR+ERD)} \pm 0.55\,\text {(syst)} \,\text {GeV}$ is the main result of this analysis, since it is constructed to provide the smallest uncertainty. The color reconnection and early resonance decay parts are separated from the rest of the systematic uncertainties. Because of the larger data sample used in this analysis, the statistical uncertainty is reduced with respect to the result of $m_{\mathrm {t}} =172.32\pm 0.25\,\text {(stat+JSF)} \pm 0.59\,\text {(syst)} \,\text {GeV}$ obtained at $\sqrt{s}=8\,\text {TeV}$. The new result is in good agreement with the value measured at $\sqrt{s}=8\,\text {TeV}$, where a leading-order ${\text{t}}{\bar{\text{t}}}$ simulation has been employed to calibrate the measurement, whereas an NLO simulation has been used here. The systematic uncertainty is increased with respect to the Run 1 result, because a broader set of CR models has been compared, which have become available in pythia  8.

## Combined measurement with the lepton+jets final state

This measurement is combined with the lepton+jets final state, where only electrons and muons are explicitly considered as leptons, while tau leptons enter the selection only when they decay leptonically. The corresponding analysis for the lepton+jets final state is described in Ref. [17]. All selection and analysis steps are kept unchanged. Since the same method for the mass extraction is used, a combination with the all-jets channel at the likelihood level is possible.

The total likelihood $\mathcal {L}$ is constructed from the single-channel likelihoods $\mathcal {L} _i$,

$$\begin{aligned} \mathcal {L} (m_{\mathrm {t}}, \text {JSF}) = \mathcal {L} _\text {A} (m_{\mathrm {t}}, \text {JSF}) \mathcal {L} _\text {L} (m_{\mathrm {t}}, \text {JSF}) , \end{aligned}$$

where the indices A and L indicate the all-jets and lepton+jets channel, respectively.

**Fig. 4:**
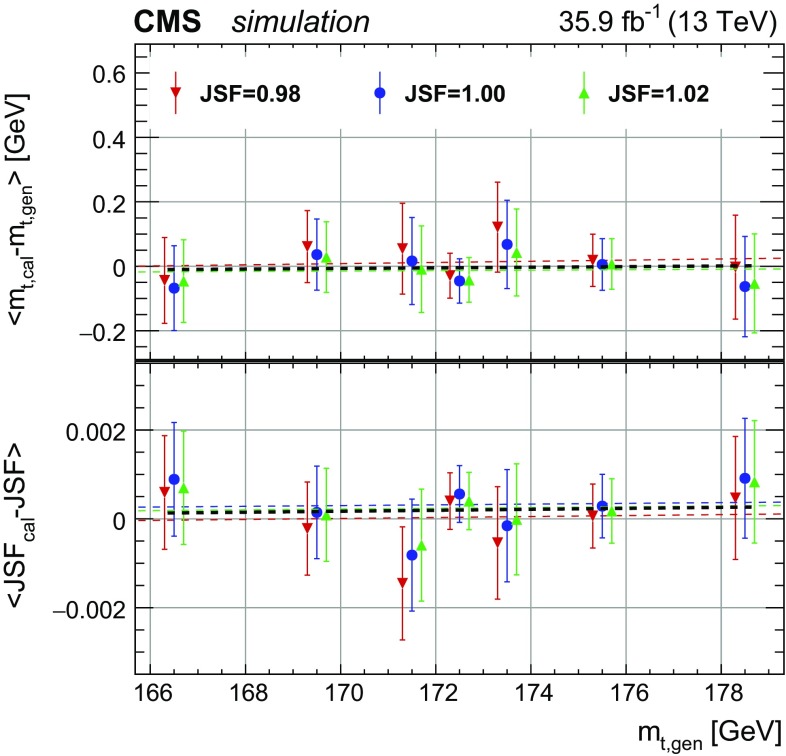
Difference between extracted and generated top quark masses (upper panel) and JSFs (lower panel) for different input masses and JSFs after the single-channel calibrations for the combined measurement. The values are extracted using the 2D method.

No extra calibration of the mass extraction is performed, but the single-channel calibrations are applied. Figure 4 shows the extracted values for the top quark mass and JSF for different input values as a validation. No residual dependence is observed.

The systematic uncertainties are evaluated as described above for the all-jets channel. For the pseudo-experiments, the systematic uncertainty sources are varied simultaneously for both channels. An exception are uncertainties that only affect a single channel. These uncertainty sources are only varied for the corresponding channel. For the all-jets channel, these are the background and trigger uncertainties. In addition, uncertainties specific to the lepton+jets channel are introduced, including the background and trigger uncertainties, as well as the uncertainties arising from the lepton isolation and identification criteria, and are described in Ref. [17]. The complete list of uncertainties is shown in Table 2. A comparison of the hybrid mass uncertainties can be found in Table 3 for the all-jets and lepton+jets channels as well as for the combination. In general, the uncertainties for the combination are smaller than those for the all-jets channel and are close to the lepton+jets uncertainties, as expected because the combination is dominated by this channel. The total uncertainty for the combination is slightly smaller than that for the lepton+jets channel.

**Table 2:** List of systematic uncertainties for the combined mass extraction. The signs of the shifts ($\delta x = x_\text {variation} - x_\text {nominal}$) correspond to the $+1$ standard deviation variation of the systematic uncertainty source. For linear sums of the uncertainty groups, the relative signs have been considered. Shifts determined using dedicated samples for the systematic variation are displayed with the corresponding statistical uncertainty

	2D		1D	Hybrid	
	$\delta m_{\mathrm {t}} ^{\text {2D}}$	$\delta \text {JSF} ^{\text {2D}}$	$\delta m_{\mathrm {t}} ^{\text {1D}}$	$\delta m_{\mathrm {t}} ^{\text {hyb}}$	$\delta \text {JSF} ^{\text {hyb}}$
	[GeV]	[%]	[GeV]	[GeV]	[%]
*Experimental uncertainties*					
Method calibration	0.03	0.0	0.03	0.03	0.0
JEC (quad. sum)	0.12	0.2	0.82	0.17	0.3
Intercalibration	$-$ 0.01	0.0	$+$ 0.16	$+$ 0.04	+ 0.1
MPFInSitu	$-$ 0.01	0.0	$+$ 0.23	$+$ 0.07	+ 0.1
Uncorrelated	$-$ 0.12	$-$ 0.2	$+$ 0.77	$+$ 0.15	+ 0.3
Jet energy resolution	$-$ 0.18	$+$ 0.3	$+$ 0.09	$-$ 0.10	+ 0.2
$\mathrm {b}$ tagging	0.03	0.0	0.01	0.02	0.0
Pileup	$-$ 0.07	+ 0.1	$+$ 0.02	$-$ 0.05	+ 0.1
All-jets background	0.01	0.0	0.00	0.01	0.0
All-jets trigger	$+$ 0.01	0.0	0.00	$+$ 0.01	0.0
$\ell$+ jets Background	$-$ 0.02	0.0	$+$ 0.01	$-$ 0.01	0.0
$\ell$+jets Trigger	0.00	0.0	0.00	0.00	0.0
Lepton isolation	0.00	0.0	0.00	0.00	0.0
Lepton identification	0.00	0.0	0.00	0.00	0.0
*Modeling uncertainties*					
JEC flavor (linear sum)	$-$ 0.39	+ 0.1	$-$ 0.31	$-$ 0.37	+ 0.1
Light quarks (uds)	$+$ 0.11	$-$ 0.1	$-$ 0.01	$+$ 0.07	$-$ 0.1
Charm	$+$ 0.03	0.0	$-$ 0.01	$+$ 0.02	0.0
Bottom	$-$ 0.31	0.0	$-$ 0.31	$-$ 0.31	0.0
Gluon	$-$ 0.22	+ 0.3	$+$ 0.02	$-$ 0.15	+ 0.2
$\mathrm {b}$ jet modeling (quad. sum)	0.08	0.1	0.04	0.06	0.1
$\mathrm {b}$ frag. Bowler–Lund	$-$ 0.06	+ 0.1	$-$ 0.01	$-$ 0.05	0.0
$\mathrm {b}$ frag. Peterson	$-$ 0.03	0.0	0.00	$-$ 0.02	0.0
semileptonic $\mathrm {b}$ hadron decays	$-$ 0.04	0.0	$-$ 0.04	$-$ 0.04	0.0
PDF	0.01	0.0	0.01	0.01	0.0
Ren. and fact. scales	0.01	0.0	0.02	0.01	0.0
ME/PS matching	$-0.10\pm \,0.08$	+ 0.1	$+\,0.02\pm \,0.05$	$+\,0.07\pm \,0.07$	+ 0.1
ME generator	$+\,0.16\pm \,0.21$	+ 0.2	$+\,0.32\pm \,0.13$	$+\,0.21\pm \,0.18$	+ 0.1
ISR PS scale	$+\,0.07\pm \,0.08$	+ 0.1	$+\,0.10\pm \,0.05$	$+\,0.07\pm \,0.07$	0.1
FSR PS scale	$+\,0.23\pm \,0.07$	$-$ 0.4	$-0.19\pm \,0.04$	$+\,0.12\pm 0.06$	$-$ 0.3
Top quark $p_{\mathrm {T}}$	$+$ 0.01	$-$ 0.1	$-$ 0.06	$-$ 0.01	$-$ 0.1
Underlying event	$-0.06\pm \,0.07$	+ 0.1	$+\,0.00\pm \,0.05$	$-0.04\pm \,0.06$	+ 0.1
Early resonance decays	$-0.20\pm \,0.08$	+ 0.7	$+\,0.42\pm \,0.05$	$-0.01\pm \,0.07$	+ 0.5
CR modeling (max. shift)	$+\,0.37\pm \,0.09$	$-$ 0.2	$+\,0.22\pm \,0.06$	$+\,0.33\pm \,0.07$	$-$ 0.1
“gluon move” (ERD on)	$+\,0.37\pm \,0.09$	$-$ 0.2	$+\,0.22\pm \,0.06$	$+\,0.33\pm \,0.07$	$-$ 0.1
“QCD inspired” (ERD on)	$-0.11\pm \,0.09$	$-$ 0.1	$-0.21\pm \,0.06$	$-0.14\pm \,0.07$	$-$ 0.1
Total systematic	0.71	1.0	1.07	0.61	0.7
Statistical (expected)	0.08	0.1	0.05	0.07	0.1
Total (expected)	0.72	1.0	1.08	0.61	0.7

**Table 3:** Comparison of the hybrid mass uncertainties for the all-jets and lepton+jets [17] channels, as well as the combination. The signs of the shifts follow the convention of Tables 1 and 2

	$$\delta m_{\mathrm {t}} ^{\text {hyb}}~\text {[GeV]}$$		
	All-jets	$\ell$+jets	Combination
*Experimental uncertainties*			
Method calibration	0.06	0.05	0.03
JEC (quad. sum)	0.15	0.18	0.17
Intercalibration	$-$ 0.04	$+$ 0.04	+ 0.04
MPFInSitu	$+$ 0.08	$+$ 0.07	+ 0.07
Uncorrelated	$+$ 0.12	$+$ 0.16	+ 0.15
Jet energy resolution	$-$ 0.04	$-$ 0.12	$-$ 0.10
$\mathrm {b}$ tagging	0.02	0.03	0.02
Pileup	$-$ 0.04	$-$ 0.05	$-$ 0.05
All-jets background	0.07	$-$	0.01
All-jets trigger	$+$ 0.02	$-$	+ 0.01
$\ell$+jets background	–	$+$ 0.02	$-$ 0.01
*Modeling uncertainties*			
JEC flavor (linear sum)	$-$ 0.34	$-$ 0.39	$-$ 0.37
light quarks (uds)	$+$ 0.07	$+$ 0.06	+ 0.07
charm	$+$ 0.02	$+$ 0.01	+ 0.02
bottom	$-$ 0.29	$-$ 0.32	$-$ 0.31
gluon	$-$ 0.13	$-$ 0.15	$-$ 0.15
$\mathrm {b}$ jet modeling (quad. sum)	0.09	0.12	0.06
$\mathrm {b}$ frag. Bowler–Lund	$-$ 0.07	$-$ 0.05	$-$ 0.05
$\mathrm {b}$ frag. Peterson	$-$ 0.05	$+$ 0.04	$-$ 0.02
semileptonic $\mathrm {b}$ hadron decays	$-$ 0.03	$+$ 0.10	$-$ 0.04
PDF	0.01	0.02	0.01
Ren. and fact. scales	0.04	0.01	0.01
ME/PS matching	$+$ 0.24	$-$ 0.07	+ 0.07
ME generator	$-$	$+$ 0.20	+ 0.21
ISR PS scale	$+$ 0.14	$+$ 0.07	+ 0.07
FSR PS scale	$+$ 0.18	$+$ 0.13	+ 0.12
Top quark $p_{\mathrm {T}}$	$+$ 0.03	$-$ 0.01	$-$ 0.01
Underlying event	$+$ 0.17	$-$ 0.07	$-$ 0.06
Early resonance decays	$+$ 0.24	$-$ 0.07	$-$ 0.07
CR modeling (max. shift)	$-$ 0.36	$+$ 0.31	+ 0.33
“gluon move” (ERD on)	$+$ 0.32	$+$ 0.31	+ 0.33
“QCD inspired” (ERD on)	$-$ 0.36	$-$ 0.13	$-$ 0.14
Total systematic	0.70	0.62	0.61
Statistical (expected)	0.20	0.08	0.07
Total (expected)	0.72	0.63	0.61

**Fig. 5:**
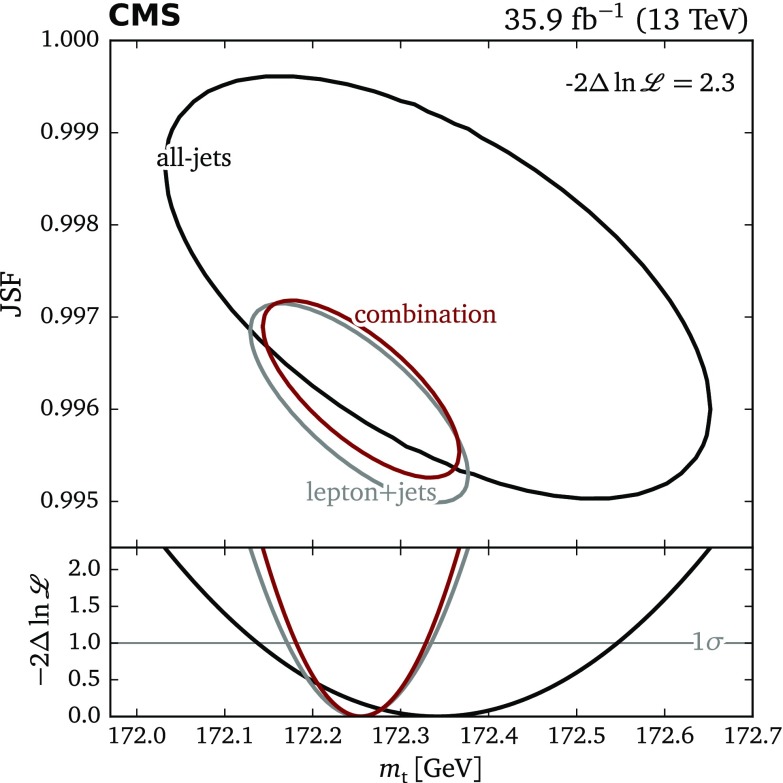
Likelihood contours for $-2\varDelta \ln \mathcal {L} =2.3$, corresponding to the $68\%$ confidence level, in the $m_{\mathrm {t}}$-$\text {JSF}$ plane (upper panel) and the likelihood profiles for the top quark mass (lower panel), where the level corresponding to one standard deviation ($\sigma$) is indicated. The hybrid measurement results for the all-jets and lepton+jets channels, as well as for the combination, are shown

The combined measurement yields

$$\begin{aligned} m_{\mathrm {t}}^{\text {2D}}&= 172.39\pm \,0.08\,{\text {(stat+JSF)}} \pm 0.71{\text { (syst) GeV and }} \\ {\text {JSF}}^{{\text{2D}}}&= 0.995\pm 0.001{\text{ (stat)}} \pm 0.010\,{\text{(syst)}} \end{aligned}$$

for the 2D method and

$$\begin{aligned} m_{\mathrm {t}}^{\text{1D}}&= 171.94\pm \,0.05\,{\text {(stat)}} \pm 1.07 {\text{ (syst)  GeV}} \\ m_{\mathrm {t}} ^{\text{hyb}}&= 172.26\pm 0.07{\text { (stat+JSF)}} \pm 0.61 {\text{ (syst)  GeV,  and }} \\ {\text {JSF}}^{{\text {hyb}}}&= 0.996\pm 0.001{\text { (stat)}} \pm 0.007{\text { (syst)}} \end{aligned}$$

for the 1D and hybrid fits. The likelihood contours for $-2\varDelta \ln \mathcal {L} =2.3$, corresponding to the $68\%$ confidence level, in the $m_{\mathrm {t}}$-$\text {JSF}$ plane are shown in Fig. 5 for the hybrid measurement results for the all-jets and lepton+jets channels, as well as for the combination. Additionally, the likelihood profiles are displayed as a function of $m_{\mathrm {t}}$. Both channels are in statistical agreement with each other. The result of the combination is closer to the lepton+jets channel, as expected.

Just as for the single-channel results, the hybrid measurement provides the best precision and is considered the main result. This is the first top quark mass measurement using the ${\mathrm {t}\overline{\mathrm {t}}}$ lepton+jets and all-jets final states combined in a single likelihood function. The largest uncertainty contribution is related to the modeling of color reconnection, as it was observed for the all-jets channel and the lepton+jets channel before using the same CR models. Accordingly, the quoted systematic uncertainty is larger than those reported in the most precise combination reported by the CMS Collaboration [12], and comparable to the value reported by the ATLAS Collaboration [50].

## Summary

A measurement of the top quark mass ($m_{\mathrm {t}}$) using the all-jets final state is presented. The analyzed data set was collected by the CMS experiment in proton–proton collisions at $\sqrt{s}=13\,\,\text {TeV}$ that correspond to an integrated luminosity of $35.9{\,\text {fb}^{-1}}$. The kinematic properties in each event are reconstructed using a constrained fit that assumes a ${\mathrm {t}\overline{\mathrm {t}}}$ hypothesis, which suppresses the dominant multijet background and improves the mass resolution.

The value of $m_{\mathrm {t}}$ and an additional jet energy scale factor ($\text {JSF}$) are extracted using the ideogram method, which uses the likelihood of the values of $m_{\mathrm {t}}$ and $\text {JSF}$ in each event to determine these parameters. The resulting $m_{\mathrm {t}}$ is measured to be $172.34\pm 0.20\,\text {(stat+JSF)} \pm 0.70\,\text {(syst)} \,\text {GeV}$. This is in good agreement with previous CMS results obtained at $\sqrt{s} = 7$, 8, and $13\,\text {TeV}$. The modeling uncertainties are larger than in the previous measurements at lower center-of-mass energies because of the use of new alternative color reconnection models that were not previously available.

A combined measurement using also the lepton+jets final state results in $m_{\mathrm {t}} = 172.26\pm 0.07\,\text {(stat+JSF)} \pm 0.61\,\text {(syst)} \,\text {GeV}$. This is the first combined $m_{\mathrm {t}}$ result obtained in the all-jets and lepton+jets final states using a single likelihood function.

## Data Availability

This manuscript has no associated data or the data will not be deposited. [Authors’ comment: Release and preservation of data used by the CMS Collaboration as the basis for publications is guided by the CMS policy as written in its document “CMS data preservation, re-use and open access policy” (https://cms-docdb.cern.ch/cgi-bin/PublicDocDB/RetrieveFile?docid=6032&filename=CMSDataPolicyV1.2.pdf&version=2).]
